# A clinical decision support system for AI-assisted decision-making in response-adaptive radiotherapy (ARCliDS)

**DOI:** 10.1038/s41598-023-32032-6

**Published:** 2023-03-31

**Authors:** Dipesh Niraula, Wenbo Sun, Jionghua Jin, Ivo D. Dinov, Kyle Cuneo, Jamalina Jamaluddin, Martha M. Matuszak, Yi Luo, Theodore S. Lawrence, Shruti Jolly, Randall K. Ten Haken, Issam El Naqa

**Affiliations:** 1 Department of Machine Learning, Moffitt Cancer Center, Tampa, FL 33612 USA; 2 University of Michigan Transport Research Institute, University of Michigan, Ann Arbor, MI 48109 USA; 3 Department of Industrial and Operations Engineering, University of Michigan, Ann Arbor, MI 48109 USA; 4 Department of Health Behavior and Biological Sciences, University of Michigan, Ann Arbor, MI 48109 USA; 5 Department of Radiation Oncology, University of Michigan, Ann Arbor, MI 48109 USA; 6 Department of Nuclear Engineering and Radiological Sciences, University of Michigan, Ann Arbor, MI 48109 USA

**Keywords:** Cancer, Software, Cancer

## Abstract

Involvement of many variables, uncertainty in treatment response, and inter-patient heterogeneity challenge objective decision-making in dynamic treatment regime (DTR) in oncology. Advanced machine learning analytics in conjunction with information-rich dense multi-omics data have the ability to overcome such challenges. We have developed a comprehensive artificial intelligence (AI)-based optimal decision-making framework for assisting oncologists in DTR. In this work, we demonstrate the proposed framework to Knowledge Based Response-Adaptive Radiotherapy (KBR-ART) applications by developing an interactive software tool entitled Adaptive Radiotherapy Clinical Decision Support (ARCliDS). ARCliDS is composed of two main components: Artifcial RT Environment (ARTE) and Optimal Decision Maker (ODM). ARTE is designed as a Markov decision process and modeled via supervised learning. Given a patient’s pre- and during-treatment information, ARTE can estimate treatment outcomes for a selected daily dosage value (radiation fraction size). ODM is formulated using reinforcement learning and is trained on ARTE. ODM can recommend optimal daily dosage adjustments to maximize the tumor local control probability and minimize the side effects. Graph Neural Networks (GNN) are applied to exploit the inter-feature relationships for improved modeling performance and a novel double GNN architecture is designed to avoid nonphysical treatment response. Datasets of size 117 and 292 were available from two clinical trials on adaptive RT in non-small cell lung cancer (NSCLC) patients and adaptive stereotactic body RT (SBRT) in hepatocellular carcinoma (HCC) patients, respectively. For training and validation, dense data with 297 features were available for 67 NSCLC patients and 110 features for 71 HCC patients. To increase the sample size for ODM training, we applied Generative Adversarial Networks to generate 10,000 synthetic patients. The ODM was trained on the synthetic patients and validated on the original dataset. We found that, Double GNN architecture was able to correct the nonphysical dose-response trend and improve ARCliDS recommendation. The average root mean squared difference (RMSD) between ARCliDS recommendation and reported clinical decisions using double GNNs were 0.61 [0.03] Gy/frac (mean [sem]) for adaptive RT in NSCLC patients and 2.96 [0.42] Gy/frac for adaptive SBRT HCC compared to the single GNN’s RMSDs of 0.97 [0.12] Gy/frac and 4.75 [0.16] Gy/frac, respectively. Overall, For NSCLC and HCC, ARCliDS with double GNNs was able to reproduce 36% and 50% of the good clinical decisions (local control and no side effects) and improve 74% and 30% of the bad clinical decisions, respectively. In conclusion, ARCliDS is the first web-based software dedicated to assist KBR-ART with multi-omics data. ARCliDS can learn from the reported clinical decisions and facilitate AI-assisted clinical decision-making for improving the outcomes in DTR.

## Introduction

Optimal decision-making in Knowledge Based Response-Adaptive Radiotherapy (KBR-ART) is a difficult task^1^. The difficulties arise from a slew of factors, such as, involvement of many variables, uncertainty in treatment response, and inter-patient heterogeneity^2^. In the absence of a quantitative framework, clinical decisions are primarily influenced by physician’s professional experiences, which may result in inter-physician variability. Thus, there is a need for a robust and user-friendly clinical decision-support tool for objective decision-making in KBR-ART that is data-driven and consistent^3^.

Adaptive Radiotherapy Clinical Decision Support (ARCliDS) is a web-based software tool for AI-assisted optimal decision-making in KBR-ART^4–6^ and potentially other oncology applications involving dynamic treatment regime (DTR)^7,8^. ARCliDS provides a quantitative approach to overcome the decision-making difficulties via a set of data analytics algorithms, which includes feature selection of important variables, statistical ensemble for representing uncertainties of treatment response, and, most importantly, integration of information-rich dense multi-omics datasets for capturing inter-patient heterogeneity^9–11^. ARCliDS combines all the above data analytics capabilities and presents a user-friendly interface for evaluating relevant clinical use cases. Moreover, it is complementary to the current treatment planning system; the integration may facilitate an introduction of multi-omics information into the treatment planning workflow.

**Figure 1:**
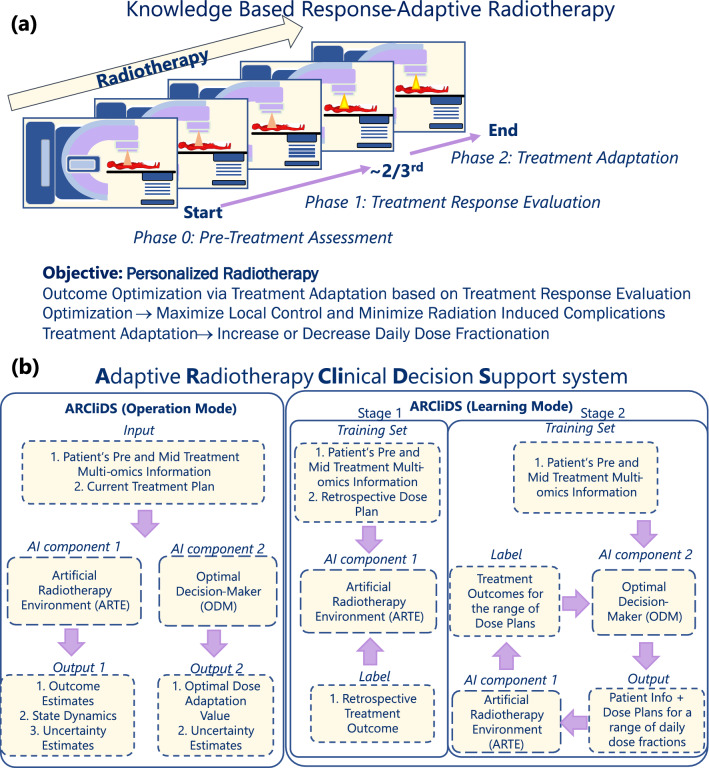
**(a)** Knowledge Based Response-Adaptive Radiotherapy (KBR-ART). In KBR-ART, a pre-treatment assessment is conducted in phase 0 and appropriate treatment plan is tailored. Then patients’ treatment response is evaluated in Phase 1, and an optimal treatment adaptation is planned and executed in Phase 2. **(b)**. ARCliDS Blueprint. ARCliDS is composed of two AI components: (1) Artificial Radiotherapy Environment (ARTE) and (2) Optimal Decision-Maker (ODM). ARCLiDS learns ARTE via supervised learning. ARTE is then utilized in planning and teaching ODM via reinforcement learning. In the operation mode, ARTE outputs State Dynamics and RT Outcome estimates while ODM outputs the optimal dose adaptation recommendation. Both ARTE and ODM present uncertainty estimates. The 3D human shape image in red color is obtained from http://www.humanshape.org/ and printed with the developer’s permission under a CC BY open access license.

DTR including adaptive RT (ART)^12^ are designed for treatment personalization. A popular ART paradigm and implementation is to adapt treatment plans to accommodate during-treatment anatomical changes due to weight loss, tumor regression and/or diminution of the volume of surrounding normal tissue and organ at risk (OAR). A complementary ART paradigm is KBR-ART which provides a response-based adaptive framework for personalizing RT as shown in Fig. 1a, where the response assessment is not limited to observing anatomical changes. It is divided into three phases: Pre-Treatment Assessment, Treatment Response Evaluation (evaluation phase) and Treatment Adaptation (adaptation phase). In the pre-treatment phase, a patient’s disease and condition is assessed and a treatment plan is tailored. In the evaluation phase, a patient’s treatment response is evaluated by comparing pre and mid treatment multi-omics information changes. Based on the treatment responses, the patient’s associated outcome probabilities are estimated. In the adaptation phase, treatment planning is adapted for a personalized and an optimal outcome. Two endpoints are considered: tumor control and normal tissue complication. The goal of KBR-ART is to maximize tumor control probability (TCP) and minimize normal tissue complication probability (NTCP).

To demonstrate the potential of ARCliDS, two clinical use cases are presented. In both studies, the evaluation time was around 1 month. The first use case is based on the UMCC (University of Michigan Cancer Center) 2007-123 phase II dose escalation clinical trial NCT01190527^13^, where inoperable or unresectable non-small cell lung cancer (NSCLC) patients were administered with 30 daily dose fractions. The patients received roughly 50 Gy [Gray = J/Kg] equivalent dose in 2 Gy fractions (EQD2) in the evaluation phase and up to a total dose of 92 Gy EQD2 in the adaptation phase. The evaluation phase lasted for roughly two-thirds of the 6-week treatment period. In the second clinical use case, patients with hepatocellular carcinoma (HCC) received adaptive SBRT in clinical trials NCT01519219, NCT01522937, and NCT0246083514^14^. In the evaluation phase, patients received 3 daily dose fractions followed by 1 month break, and in the adaptation phase, a suitable sub-population of the patients received 2 additional daily doses.

A large sample size that is representative of the true population is preferred for all data driven and statistical modeling applications. However, due to financial, feasibility, and ethical reasons, obtaining a large dataset in medical field is often impractical. In our case, a dataset of size 117 and 292 were available for NSCLC patients and HCC patients, respectively. Dense multi-omics data with 297 features were available for only 67 NSCLC patients and 110 features for 71 HCC patients. These datasets, albeit on the smaller size, are unique as KBR-ART is still in its clinical trial phase and hence among the largest multi-omics datasets for KBR-ART. The multi-omics data consists of clinical, dosimetric, radiomics, and molecular biomarkers, such as genomics (single nucleotide polymorphisms), transcriptomics (micro-RNA), and proteomics (cytokines).

Under the current United States Food and Drug Administration (FDA) definition and guidelines, ARCliDS is categorized as a Software as a Medical Device (SaMD)^6^. SaMD is defined as software intended to be used for medical purposes independently in contrast to software intended to drive a hardware medical device (software in a medical device). This definition was recently adopted by FDA to include AI software^15,16^ which can automatically learn from user cases and continuously update after deployment, as opposed to traditional software, which stays fixed after deployment (excluding version update). Therefore, ARCliDS also has two modes of operation: Operation Mode and Learning Mode as shown in Fig. 1b. After the initial training, both modes can run simultaneously (online learning) in the clinic.

ARCliDS is composed of two main AI components. The first component is the Artificial Radiotherapy Environment (ARTE) for estimating the predicted outcome and the second component is the Optimal Decision-Maker (ODM) for decision-making. In Operation Mode, ARCliDS asks for a patient’s pre and mid treatment multi-omics information, and current treatment plan. It feeds that information into ARTE and ODM, and obtains outcome estimates, state dynamics, and the optimal dose adaptation value. All of the estimated results come with associated uncertainty. The results are presented in two main plots: outcome space spanned by TCP and NTCP, and population distribution plots as further explained in the Graphical User Interface (GUI). During the Learning Mode, ARTE is trained first on the available data, and then ODM is trained on the ARTE. The details of the training are presented in the “Methods” section and Supplementary Materials (SM).

ARCliDS presents a significant improvement to Tseng et al.^17^ and Niraula et al.^5^ methods. The improvement comes from the graphical representation of patients’ features. Convolution neural networks (CNNs) are known to perform well because they exploit the feature locality of images^18^. In other words, pixels at neighboring areas of an image are correlated, and CNN architectures can capture those correlations. Graph Neural Networks (GNNs) are similar except they can also exploit the non-local relationship between feature values^19,20^. Computationally, GNNs use fewer network connections compared to fully connected NNs, which help in learning by reducing redundancies. From another perspective, information from one feature only goes to its neighboring features. In this work, we have borrowed the feature graph from Luo et al.’s work on multi-objective Bayesian Network^21^ which identified the most important features related to RT outcome of interest by finding the Markov Blanket of the outcomes. Details of feature selection procedure are presented in SM Sect. S1. For both NSCLC and HCC, we were able to (coincidentally) select 13 important features.

The important highlights of our work as as follows. ARTE is composed of transition function (TF), RT outcome estimator (RTOE) and Reward Function (RF). To define patients state, we carried out feature selection via multi-objective Markov Blanket feature selection process. In this work, we have proposed a linear-quadratic-linear (LQL) type TF. This is an improvement from Niraula et al.^5^ work which employed a linear-quadratic (LQ) type function. LQL model is a generalization of LQ model that covers both RT and SBRT. For RTOE, we applied GNN to general logistic function guided double NN architecture, which was developed by Niraula et al.^5^ To improve the robustness of ODM, we have replaced the online learning approach with the Planning and Learning approach^22^. We trained and validated ARCliDS in two different cancer sites and different treatment types to show its versatility. For robust validation of ARTE, we performed 10-fold stratified shuffle cross validation. For the robust validation of ODM, we trained ODM on synthetic dataset generated via Generative Adversarial Network (GAN) and then evaluated on the original dataset, which was repeated for a total of 5 times to report the deviation. For baseline comparison, we trained, evaluated, and then compared ARCliDS built with single GNN and general logistic function guided double-GNN (GloGD-GNN) RTOEs.

## Results

### Selected multi-omics features for TCP and NTCP

Thirteen important multi-omics features resulted from the multi-objective Markov Blanket feature selection process. These features are important predictors for both TCP and NTCP. For NSCLC, the selected features are *cytokines*: pretreatment interleukin 4 (pre-IL4), pre-IL15 and slope of Interferon gamma-induced protein 10 (slope-IP10); *Tumor PET imaging features/Radiomics*: pretreatment Metabolic Tumor Volume (pre-MTV), relative difference (RD) of Gray-level size zone matrices (GLSZM)-large zone low gray-level (LZLGE) and RD-GLSZM-zone size variance (RD-GLSZM-ZSV); *Dosimetry*: Tumor gEUD and Lung gEUD; *Genetics (single nucleotide polymorphism [SNP])*: Cxcr1- Rs2234671, Ercc2-Rs238406, and Ercc5-Rs1047768; and MicroRNA: miR-191-5p and miR-20a-5p. A description of the features is presented in SM Table S3. Here the slope and RD were determined from comparing pre-treatment and mid-treatment or the end of evaluation phase.

For HCC, the selected features are *clinical*: sex, age, pretreatment cirrhosis status (pre-cirrhosis), pretreatment Eastern Cooperative Oncology Group Performance Status (pre-ECOG-PS), number of active liver lesions (active lesions), pretreatment albumin level (pre-albumin); *Tumor PET Imaging*: gross tumor volume (GTV) and liver volume minus GTV (Liver-GTV); *Dosimetry*: GTV gEUD and Liver-GTV volume; and *cytokines/signaling molecule*: relative difference of Transforming growth factor beta (RD-TGF-$\beta$), Cluster of Differentiation 40 receptor’s Ligand (RD-CD40L), and Hepatocyte growth factor (RD-HGF). A description of the features is presented in SM Table S11.

### ARTE with GLoGD-GNN architecture yielded expected monotonic dose-response

As expected, correction of RTOE with GLoGD architecture helps to maintain the monotonic relationship between the outcome probability and daily dose fractionation. An example is provided in Figs. S7, S8, S20, and S21. We performed the area under the receiver operating characteristics curve (AUROCC) analysis to measure the performance of single GNN and GLoGD GNN architecture. For the analysis, we split the data set via a 10-fold stratified shuffle 80–20 split process. For NSCLC, the single-GNN validation set AUROCC for TCP and NTCP were $0.77 \pm 0.14$ (mean ± SD) and $0.73 \pm 0.18$, while GLoGD-AUROCC for TCP and NTCP were $0.73 \pm 0.15$ (mean ± SD) and $0.79 \pm 0.17$, respectively. For HCC, the single-GNN AUROCC for TCP and NTCP were $0.72\pm 0.31$ (mean ± SD) and $0.81\pm 0.14$, while GLoGD-AUROCC for TCP and NTCP were $0.74 \pm 0.27$ (mean ± SD) and $0.68\pm 0.24$, respectively.

As a further evaluation, we calculated Youden’s J score from the training set, obtained the optimal threshold, and then found the sensitivity and specificity of the validation set. For NSCLC, the single-GNN validation set optimal threshold, sensitivity and specificity for TCP were $0.44 \pm 0.06$, $0.73 \pm 0.19$ and $0.63 \pm 0.24$, and for NTCP were $0.56 \pm 0.04$, $0.73 \pm 0.26$ and $0.68 \pm 0.18$, while the GloGD-GNN metrics in the same order for TCP were $0.59 \pm 0.16$, $0.58 \pm 0.31$, $0.58 \pm 0.26$ and for NTCP were $0.61 \pm 0.09$, $0.50 \pm 0.28$, $0.78 \pm 0.18$, respectively. For HCC, the single-GNN validation set optimal threshold, sensitivity and specificity for TCP were $0.64 \pm 0.46$, $0.94 \pm 0.09$ and $0.60 \pm 0.52$, and for NTCP were $0.90 \pm 0.24$, $0.10 \pm 0.21$ and $0.91 \pm 0.12$, while the GloGD-GNN metrics in the same order for TCP were $0.75 \pm 0.23$, $0.39 \pm 0.45$, $0.70 \pm 0.48$ and for NTCP were $0.59 \pm 0.16$, $0.25 \pm 0.35$, $0.69 \pm 0.31$, respectively. The performance of GLoGD-GNN on the population dataset is either similar or poor compared to single-GNN because GLoGD-GNN is designed for individual patient.

### Generating synthetic patients via GAN for training ODM

To extend the sample size of our dataset for training ODM, we trained Wasserstein Generative Adversarial Network with Gradient Penalty (WGAN-GP)^5,23^ on the original dataset and generated 10,000 synthetic patients. GAN can learn the underlying population distribution in the multi-dimensional feature space. We compared the distribution of synthetic patient with the original patient population data using the Jensen Shannon Divergence (JSD). JSD value of 0 means complete overlap and 1 means complete separation. JSD for NSCLC’s selected features were pre-IL4: 0.30, pre-IL15: 0.18, slope-IP10: 0.50, Pre-MTV: 0.46, RD-GLSZM-LZLGE: 0.59, RD-GLSZM-ZSV: 0.56, Tumor-gEUD: 0.60, Lung-gEUD: 0.43, miR-191-5p: 0.18, miR-20a-5p: 0.18, cxcr1-Rs2234671: 0.16, errc2-Rs238406: 0.24, and ercc5-Rs1047768: 0.07. JSD for HCC’s selected features were sex: 0.03, age: 0.44, pre-cirrhosis: 0.25, active lesion: 0.31, GTV: 0.33, Liver-GTV: 0.36, pre-ECOG-PS: 0.16, pre-albumin: 0.46, RD-TGF-$\beta$: 0.47, RD-CD40L: 0.45, RD-HGF: 0.75, GTV-gEUD: 0.45, and Liver-GTV gEUD: 0.45. Note that, we did not perform any statistical hypothesis test on the learned distribution as it is not necessary for the training of ODM. In principle, a uniform distribution works just as fine, however, with increase in computational complexity. Nevertheless, we additionally ascertained the similarity by visual inspection as shown in SM Figs. S13 and S26.

### Evaluation of ARCliDS recommendation

ARCliDS was trained and validated on two different use cases from two different types of RT treatments. The first use case is an adaptive RT clinical trial of NSCLC patients, and the second use case is an adaptive SBRT clinical trial. After feature selections, we first built five ARTEs for each disease using the dataset. We then generated 10,000 synthetic patients using a generative adversarial network (GAN)^23^. Five ODMs were trained using the five ARTEs and 4000 randomly chosen patients from the pool of the 10,000 synthetic patients. The trained ODM models were then validated on the original dataset.

Since there is no ground truth of what an optimal radiation dosage for a certain outcome would be, our evaluations are based on two metrics. The first metric is root mean square difference (RMSD) value between the ODM recommendation and the retrospective clinical decision used in treatment planning. However, since RMSD is a symmetric metric, i.e., it cannot differentiate a higher dose from a lower dose recommendation compared to the clinical decision, we separated the positive and negative clinical outcomes for additional insight. For the positive clinical outcome, a lower RMSD indicates agreement with the good clinical decisions. For the bad clinical outcome, additional comparison is needed. For this purpose, we have adopted a second metric for self-evaluation as presented in Table 1. The self-evaluation scheme is also based on the assumption that increasing radiation results in a higher value for both TCP and NTCP. Using this assumption, we can further evaluate the recommendations for patients with negative clinical outcome.

**Table 1:** Self-Evaluation scheme.

TC	NTC	Relation	Remark
Positive clinical outcome			
1	0	$|Al - Cl| \le 10\%$ of $D_{max}$	Good
1	0	$|Al - Cl| > 10\%$ of $D_{max}$	Not sure
Negative clinical outcome			
0	0	$Al \le Cl$	Bad
0	0	$Al > Cl$	Good
0	1	$Al < Cl$	Good
0	1	$Al \ge Cl$	Bad
1	1	$Al < Cl$	Good
1	1	$Al \ge Cl$	Bad

Evaluation scheme for AI recommendation is based on the positive relation between radiation dose and treatment outcomes, i.e., both TCP and NTCP increases with an increase in radiation dose. Here TC and NTC are clinical treatment outcome. TC = 1 and NTC = 0 are the only clinically positive outcome. For a patient with known treatment outcome, we can evaluate an AI recommendation by comparing it with the retrospective clinical decision. For instance, for a patient with TC = 0 and NTC = 0, a higher dose recommendation is good, while for a patient with TC = 1 and NTC = 1, a lower dose recommendation is good, and for a patient with TC=0, NTC=1, a lower dose recommendation is good. For the clinically positive cases, we cannot judge for sure if a recommendation is good unless it is within a window of the clinical dose decision. We have set the window to be 10% of the maximum dose used in the modeling.

*Al* ARCliDS recommendation, *Cl* clinical decision, *TC* tumor control, *NTC* radiation-induced normal tissue complication, $D_{max}$ maximum dose value used in modeling, 0, no event; 1, event.

Additionally, we present a comparison for two different ARCliDS models. The first is built with Single GNN as RTOE and fully connected double deep Q-network as ODM (Single GNN RTOE+ DDQN ODM) and the second with GLoGD GNN as RTOE and fully connected double deep Q- network as ODM (GLoGD GNN RTOE + DDQN ODM).

**Figure 2:**
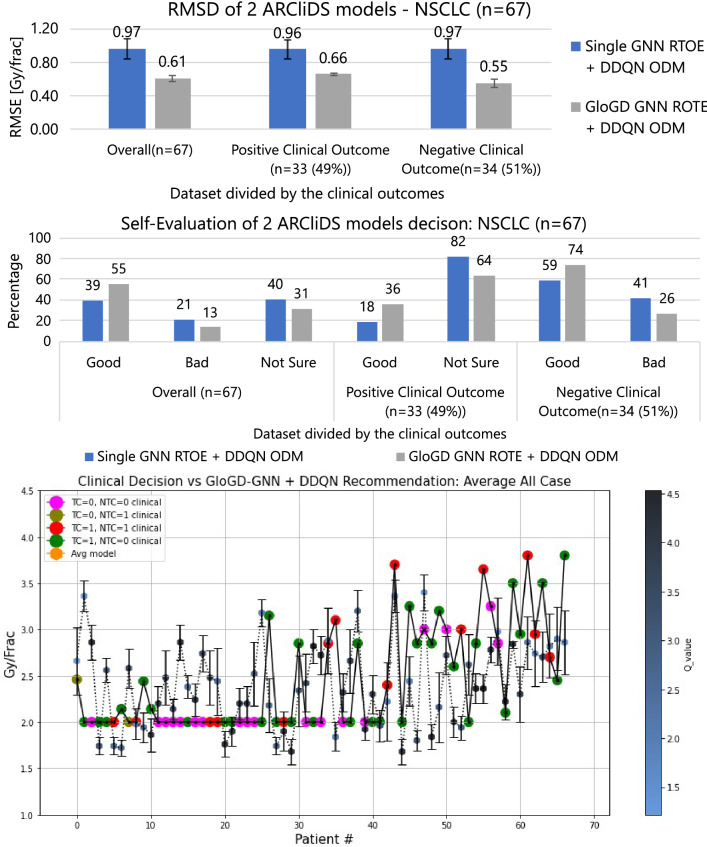
Comparison and analysis of 2 ARCliDS models trained and validated on Adaptive RT NSCLC patients. The top bar diagram presents RMSD, the middle bar diagram presents Self-Evaluation, and the bottom plot presents a visual comparison between the ARCliDS recommendation and clinical decision. The clinical decisions are color coded with the outcomes and the ARCliDS recommendations are color coded with the respective q-value. Qualitatively, the q-value can be considered as the AI confidence in its recommendations.

**Figure 3:**
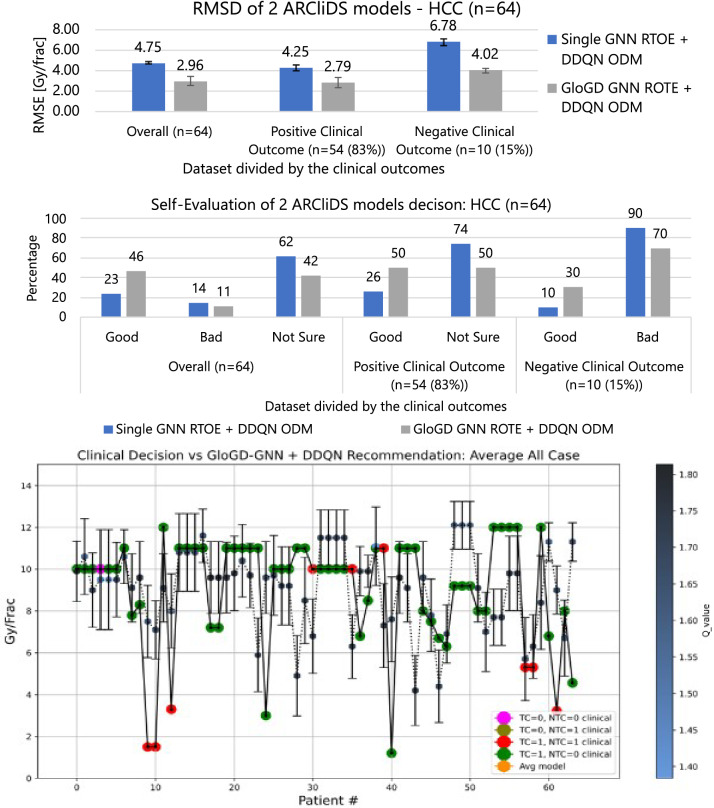
Comparison and analysis of 2 ARCliDS models trained and validated on Adaptive RT HCC patients. The top bar diagram presents RMSD, the middle bar diagram presents Self-Evaluation, and the bottom plot presents a visual comparison between the ARCliDS recommendation and clinical decision. The clinical decisions are color coded with the outcomes and the ARCliDS recommendations are color coded with the respective q-value. Qualitatively, the q-value can be considered as the AI confidence in its recommendations.

#### RMSD evaluation of ARCliDS recommendation

The ODM was trained on the synthetic dataset using the trained ARTE and validated on the original dataset. The main results are summarized and presented in Figs. 2 and 3 and in SM Sects. S7.5 and S8.5. For the NSCLC patients, the overall RMSDs between the two ARCliDS models’ average recommendation and reported clinical decisions, ordered as GLoGD GNN RTOE + DDQN ODM and Single GNN ROTE +DDQN ODM, were $0.61\pm 0.03$ Gray/fraction [Gy/frac] (mean±sem) versus $0.97\pm 0.12$ Gy/frac, respectively. The RMSDs for patients with positive clinical outcomes were $0.66\pm 0.02$ Gy/frac versus $0.96\pm 0.11$ Gy/frac respectively, and for patients with negative clinical outcomes were $0.55\pm 0.05$ Gy/frac versus $0.97\pm 0.12$ Gy/frac, respectively.

For the HCC patients, the overall RMSDs were $2.96\pm 0.42$ Gy/frac versus $4.75\pm 0.16$ Gy/frac, respectively. The RMSD for patients with positive clinical outcomes were $2.79\pm 0.50$ Gy/frac versus $4.25\pm 0.26$ Gy/frac, respectively, and for patients with negative clinical outcomes were $4.02\pm 0.23$ Gy/frac versus $6.78\pm 0.35$ Gy/frac, respectively.

#### Self-evaluation of ARCliDS recommendation

For the NSCLC patients, the overall Self-Evaluation results between the two ARCliDS models’ average recommendation and reported clinical decisions, ordered as GLoGD GNN RTOE + DDQN ODM and Single GNN ROTE +DDQN ODM, were Good: 55% versus 39%, Bad: 13% versus 21%, and Not Sure: 13% versus 40%, respectively. The Self-Evaluation results for patients with positive clinical outcomes, were Good: 36% versus 18%, and Not Sure: 82% versus 64%, respectively, and for patients with negative clinical outcomes were Good: 74% versus 59%, and Bad: 26% versus 41%, respectively.

For the HCC patients, the overall Self-Evaluation results were Good: 46% versus 23%, Bad: 11% versus 14%, and Not Sure: 42% versus 62%, respectively. The Self-Evaluation results for patients with positive clinical outcomes were Good: 50% versus 26%, and Not Sure: 50% versus 74%, respectively, and for patients with negative clinical outcomes were Good: 30% versus 10%, and Bad: 70% versus 90%, respectively.

## Discussions

To our knowledge, there are other public and commercial software for ART^24,25^, but ARCliDS is the first interactive software dedicated to KBR-ART that will be available through a web portal. In this work, we have shown its applicability to adaptive RT and SBRT. However, ARCliD’s underlying technology can be generalized to any other DTR to optimize sequential decision-making with multi-omics data for deciding the order of treatments, including multi-modality treatment, given that an artificial treatment environment can be sufficiently modeled.

We have implemented tools such as GAN and GNN and invented novel techniques such as GLoGD-GNN to overcome data-related issues for developing ARCliDS. We applied GAN to learn the underlying patient’s feature distribution and generated 10,000 synthetic patients for training the ODM. We adopted GNN for modeling RTOE as exploiting the inter-relationship between the features can improve model prediction. Mathematically, the inter-relationship can be represented by a directed graph *G*(*V*, *E*) where the nodes *V* represent patient features and edges E represent the relationships. Analyzing the inter-feature relationships before feeding it to the NN reduces the number of connections and hence simplify the learning process. As a novel approach, we applied GNN on the feature space as opposed to the sample space. As shown in the SM, every patient is represented by a directed graph of features, set by the treatment and disease type. RTOE is then designed as a graph classification problem where the node value differs from patient to patient.

As seen from Figs. 2, 3, the models in descending order according to the RMSD and Self-Evaluation measures, for both NSCLC and HCC, are GLoGD GNN RTOE + DDQN ODM, and Single GNN RTOE +DDQN ODM. As expected, correction of RTOE with GLoGD architecture helps to maintain the monotonic relationship between the outcome probability and daily dose fractionation and in turn helped ARCliDS in making better recommendation.

Our framework has some limitations. Clinically, RT dose adaptation can be performed in different ways: (1) change dose per fractions, and (2) change the number of fractions. For SBRT, the former is suitable, however for some diseases and modalities the latter may be more appropriate. For instance, when RT is combined with chemotherapy, increasing the number of fractions is preferred. Our framework only covers the former. ARCliDS uses several biomarkers such as cytokines as predictors. Due to the lack of standardization, biomarker levels of the same blood sample measured in two labs can be quite different also known as batch effect. So, biomarker levels of external dataset must be carefully examined before applying ARCliDS. For dosimetric predictor, we have used gEUD, however, for lung and liver, mean dose could also be applied. Another limitation is the number of NTCPs considered in ARCliDS. In practice there may be more than 1 normal tissues of interest. For NSCLC, heart and lungs are the dose-limiting organs at risk (OAR). For HCC, although liver is usually the main OAR, in some patients, who had tumors near the intestine, the intestine is also considered during designing the treatment plan. Finally, beside data-related shortcomings, ARCliDS prediction and recommendation uncertainty, which is based on statistical ensembles, can be improved by training more models; however, this will require more computational power and time. Although we have the largest dataset of its kind, a larger sample size and balanced dataset will improve ARCliDS performance. We dedicate subsequent paragraphs to discuss data-related limitations, methods we implemented to overcome those limitations, and other possible solutions.

The learning of an environment model is the bottle neck of ARCliDS. For learning a good ARTE, a sufficient sample size and a balanced dataset are necessary. In the adaptive HCC patient’s cohort, only 1 patient did not achieve local control. As a result, RTOE for TCP had an unusually high AUROCC uncertainty. Although we applied class-imbalance correction techniques such as SMOTE and weighted loss function, we witnessed that such techniques fall short in correcting a highly imbalanced dataset. In addition, the toxicity count was also low—there were only 7 patients that showed toxicity. While this is a clinically desirable result, it hinders the learning process and hampers model generalizability. To make the matter worse, patients with highest liver gEUD didn’t show toxicity as shown in Fig. S19. This reflects inter-patient heterogeneity, where some patients have poor pre-treatment liver function, who are at a higher risk of toxicity for lower dose. Nevertheless, we performed a hyperparameter search for maximizing the generalizability of ARTE.

High noise-to-signal ratio due to inter-patient heterogeneity becomes even more pronounced with a small sample size. The medical field is especially doomed with a small sample size primarily due to the privacy issue^26^. Such issues make it difficult to learn correct trends in purely data-driven learning. We found that Single-GNN RTOE predicted unphysical trends between daily dose fractionation and TCP/NTCP. For correction, we applied a GLoGD-GNN architecture to infuse prior knowledge into the data-driven technique. We found that it corrects the trend and can also increase the model predictability. Alternatively, distributive learning features such as federated learning can be added to ARCliDS to overcome the small sample size issue. In federated learning approach only the model parameters are shared and data stays within the firewall of individual institutions^27^.

Sample size issues in training ODM can also be overcome by using synthetic patients. Since ODM of ARCliDS learns via model-based reinforcement learning, computationally the task of ODM is to learn ARTE. This can be considered as an interpolation problem in a continuous feature space. This problem can be tackled using brute-force by exhaustively selecting patient’s state. However, this assumes a uniform distribution which is generally not true. Therefore, we applied generative adversarial network (GAN) to learn the underlying patient’s feature distribution, as shown in SM and generated synthetic patient states for training the ODM. In principle, a conditional GAN^28^ can be applied to generate patients states distribution along with the outcome, however, a low sample size coupled with severe class-imbalance makes it impossible to correctly learn the underlying conditional probability distribution.

We found that the RMSD values for adaptive SBRT in HCC was higher than adaptive RT in NSCLC. There are three reasons for the higher RMSD value: (i) A larger range of adaptive dose values was explored for SBRT, i.e., 1 to 15 Gy/frac compared to 1.5 to 4 Gy/frac; given that the sample sizes are comparable, the datapoints for HCC are much sparser resulting in higher interpolation error; (ii) most of the patients with a clinically negative outcome for SBRT received a lower adaptive dose than the positive case; this can confuse the RTOE, which assumes higher doses results in higher TCP and NTCP; (iii) due to class-imbalance, the corrected GLoGD-GNN RTOE yielded a flatter monotonic relation than expected that did not spanned the whole probability space; we observed that the AI agent failed to satisfy a population-based goal of TCP > 90% and NTCP < 25%. So, we set the computation goal of TCP > 50% and NTCP < 50%. A smaller RMSD value can be achieved with a large sample size and well-balanced dataset.

In conclusion, we have built a user-friendly software for AI-assisted clinical decision-making and demonstrated its performance in adaptive RT. The underlying technology behind the software is generalizable to other sequential decision-making tasks in oncology. We employed GNNs to exploit the inter-feature relationship. We trained and validated our software in two different treatment types for two different diseases. We repeated the training and validation for 2 different models to test our hypothesis of improved model performance. The results confirmed our hypothesis. Statistical Ensemble was adopted to assess the model uncertainty.

## Methods

### Graphical user interface

**Figure 4:**
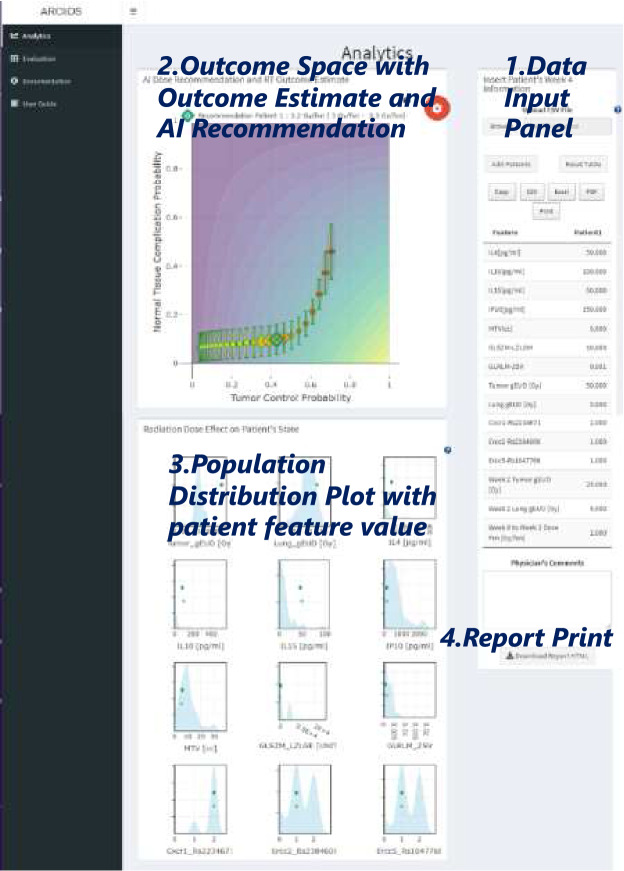
ARCliDS graphical user interface (GUI). The GUI consists of the Data Input Panel, Outcome Space spanned by TCP and NTCP, historic Population Distribution Plots, and Report Print.

We have designed ARCliDS as a Web Application (app) using R Shiny as shown in Fig. 4. The app consists of 4 main panels: Data Input Panel, Outcome Space, Population Distribution Plot, and Report Print. Beside these, there are accessibility tools such as help information, user guide, documentation, zooming, and printing option.

#### Data input panel

Patient Data can be input manually or via a data file. Multiple patients’ states can be input for visual comparison. The inputs can be saved or printed if necessary. There is a dedicated space for Physician notes.

#### Outcome space

We present the AI recommendations in the Output Space. The Output Space is spanned by TCP in the x-axis and NTCP in the y-axis. We contoured and colored it with the Reward Function, providing additional insight on the AI’s Decision Making. Given a patient’s information, it shows treatment outcome for a range of daily dose fractions and marks the treatment outcome for the optimal dose recommendation. It provides uncertainty assessment for both the outcome estimate and AI recommendation.

#### Population distribution plot

Knowing the patient’s state value and its relative position to the population, provides information on patient’s “whereabouts”. To accommodate a comparison on the feature level, we have included histograms for each feature and patient’s state value atop.

#### Report print

We have designed a report printing in html format. The interactive nature of the report, even outside the app, makes it much easier to communicate with other users.

### Artificial RT environment (ARTE)

Radiation damages both cancer cells and normal tissue cells. To quantify the relationship between the applied radiation and the treatment response, we consider the radiation absorbed by tumor and surrounding normal tissue, and the probabilities of tumor control (TCP) and normal tissue complication (NTCP).

**Figure 5:**
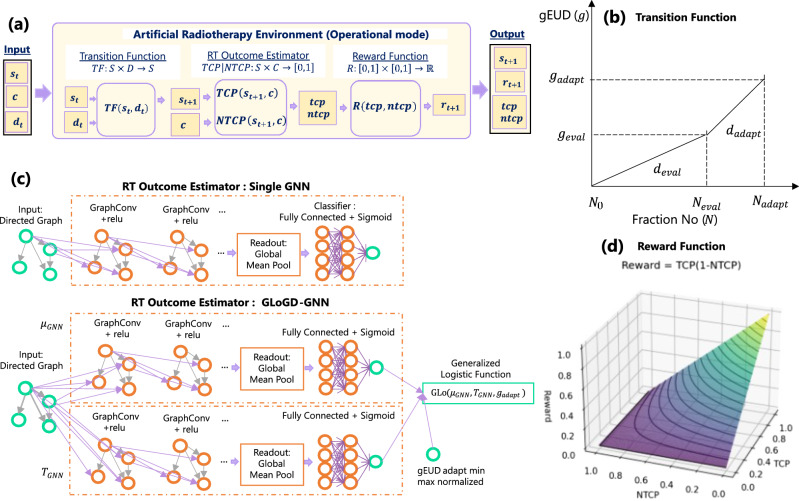
**(a)** Workflow of Artificial Radiotherapy Environment (ARTE). ARTE is composed of three functions: Transition Function (TF), RT Outcome Estimator (RTOE), and Reward Function. For patients in state $s_t$ that have been administered dose $d_t$, TF predicts the resulting state $s_{t+1}$. RTOE estimates *tcp* and *ntcp* for a patient in state $s_{t+1}$ and covariate *c*. The reward function R assigns a reward $r_{t+1}$ to the tuple (*tcp*, *ntcp*), so that optimal reward corresponds to maximal *tcp* and minimal *ntcp*. Overall, given $s_t$, *c*, and $d_t$, ARTE yields $s_{t+1}$, $r_{t+1}$, *tcp* and *ntcp*. **(b)**. Transition Function for gEUD. Here, the KBR-ART regimen is divided into three time points, pre, mid, and post treatment, denoted by the daily dose fraction, *d*, number $N_0$, $N_{eval}$, and $N_{adapt}$, respectively. The treatment period between $N_0$ and $N_{eval}$ is the Evaluation Phase and between $N_{eval}$ and $N_{adapt}$ is the Adaptation Phase. **(c)** Two GNN based RT Outcome Estimator Architectures. A Single Graph Neural Network (GNN) has an input layer for graph input, graph convolution layers for graph embedding, a global mean pool layer, and a fully connected classifier layer. Generalized logistic function guided double GNN (GLoGD-GNN) has two Single GNNs fed into a 2-parameter logistic function. GLoGD-GNN takes in gEUD as the argument. **(d)**. Reward Function 3D Contour Plot. The reward function, $r=tcp(1-ntcp)$, smoothly raises toward its maximum value at $(tcp,ntcp)=(1,0)$. AI’s goal is to find doses that will result in the maximum reward.

The absorbed radiation is spatially non-uniform, so it is generally converted to a homogeneous dose value by weighted-averaging of the treatment sites from treatment planning. Generalized equivalent uniform dose (gEUD) is one such metric^29^. It is expected that for a fixed radiation site, gEUD must increase with increasing applied radiation as shown in Fig. 5b. We have assumed a linear-quadratic-linear (LQL)^30^ type monotonic proportionality relationship (S1) as presented in the SM, which further results in the following two relationships for KBR-ART, i.e.,

1 $$\begin{aligned} {\frac{g_{adapt}-g_{eval}}{N_{adapt}-N_{eval}}} \propto {\left\{ \begin{array}{ll} d_{adapt}\left( 1+\frac{d_{adapt}}{\alpha /\beta }\right) ,&{}{\text {if}}\ d_{adapt} < D_T,\\ {D_{T} + \frac{D_{T}^{2}}{\alpha /\beta }(d_{adapt}-D_{T}),}&{}{\text {otherwise.}} \end{array}\right. } \end{aligned}$$

and,

2 $$\begin{aligned} {\frac{g_{eval}-g_{0}}{N_{eval}-N_{0}}} \propto {\left\{ \begin{array}{ll} d_{eval}\left( 1+\frac{d_{eval}}{\alpha /\beta }\right) ,&{}{\text {if}}\ d_{eval} < D_T,\\ {D_{T} + \frac{D_{T}^{2}}{\alpha /\beta }(d_{eval}-D_{T}),}&{}{\text {otherwise.}} \end{array}\right. } \end{aligned}$$

where *g* stands for gEUD, *N* for nth daily dose fractions, *d* for dose fractions, $D_T$ for threshold doses, and $\alpha /\beta$ ratio is a tissue-specific parameter. The subscript 0, *eval*, and *adapt* of *N* and *g* corresponds to pre-, mid-, and after-treatment, respectively, while $d_{eval}$ and $d_{adapt}$ corresponds to applied daily dose fractions during the evaluation phase and adaptive phase, respectively. Dividing the relationships Eqs. (1) and (2) yields four equations for $g_{adapt}$ as listed in SM Table S1.

With the assumption that the increment of radiation increases both TCP and NTCP, we have applied a sigmoid shape generalized logistic function to represent the outcome probability as follows,

3 $$\begin{aligned} p = \frac{1}{1 + \exp \left( \frac{g - \mu (s)}{T(s)}\right) } \end{aligned}$$

where the patient-specific parameters $\mu$ and *T* are functions of their multi-omics state. By applying patient’s pre and mid treatment multi-omics information, the above dose-response relationship captures inter-patient heterogeneity.

Applying the equations from Table S1 and Eq. (3), ARTE is built as a Markov Decision Process (MDP) as shown in Fig. 5a. ARTE takes in patient’s state (*s*, *c*) and daily dose fractionation (*d*) as the input and returns patient’s next state ($s^\prime$) and outcome (*tcp*, *ntcp*) as the output. The state dynamics is modeled by the TF and the associated outcome by the RTOE.

#### Patient states

Patient State, $S\subset \mathbb {R}^k$, represents a patient’s information at a given time. It consists of patient’s features such as dosimetric, clinical, radiomics, genomics, and imaging information as listed in Tables S3 and S10. MDP assumes that patient’s state at time $t+1$ only depends on patient’s state and dose, $D\subset \mathbb {R}$, at time t. In KBR-ART, three time points are relevant as shown in Fig. 5b: (i) pretreatment ($t=0$), (ii) mid treatment ($t = eval$), and (iii) post treatment ($t = adapt$).

Previously, deep regression models^5,17^ were applied to learn state transitions for all the patient features. In this work, however, to reduce modeling error, we consider the non-dosimetric variables at any time points as predictors for the treatment outcome and directly use the clinically measured values. We only estimate the state transition for dosimetric variables since the treatment outcome largely depends on the radiation. Hence, we have divided the patients states into time-varying dosimetric variables, $S\subset \mathbb {R}^m$, and a set of other fixed multi-omics covariates, $C\subset \mathbb {R}^{k-m}$. A complete patient’s state is given by $(s,c) \in S \times C$.

#### Transition function (TF)

$TF:S\times D \rightarrow S$, predicts the next state, $s_{t+1}$ for patients in state, $s_t$, under given dose, $d_{t+1}$. We have used two TFs for the dosimetric variables, tumor gEUD and normal tissue gEUD. We empirically found that deep model-based TF for gEUDs does not always maintain the causal monotonic relationship. Thus, we applied the well-known LQL-based relationship from Eq. (S1) to guarantee an increasing monotone relationship between the dose applied and dose absorbed as presented in Eqs. (1) and (2).

#### RT outcome estimator (RTOE)

RTOEs, $TCP:S\times C\rightarrow [0,1]$ and $NTCP:S\times C \rightarrow [0,1]$, estimate *tcp* and *ntcp* for the patient’s state, ($s_{t+1}, c$). In this work, we have applied GNN as the RTOE as shown in Fig. 5c. Each patient is assigned with a graph of features and then a binary classification is learned on the graph level. We first applied a single GNN for RTOE. While the performance improved drastically compared to a fully connected classifier, the single GNN was found to not respect the expected monotonicity between the dose value and the outcome probability. To meet the monotonic relationship, we applied a double GNN architecture to a generalized logistic function named as generalized logistic function guided double GNN (GLoGD-GNN).

#### Reward function (RF)

Reward function, $R:[0,1]\times [0,1]\rightarrow \mathbb {R}$, assigns a value to the (*tcp*, *ntcp*) pair. The reward function is selected such that its optimization results in maximal tcp and minimal ntcp. We have adopted, $r=tcp(1-ntcp)$, reward function for ARCliDS. As seen from Fig. 5d, it is smallest at the negative outcomes, $\{(tcp,ntcp)\}=\{(0,0),(0,1),(1,1)\}$, and largest at the positive outcome, $(tcp,ntcp)= (1,0)$.

Additionally, a goal is defined. By default, the goal can be defined as $tcp>50\%$ and $ntcp < 50\%$, which rounds to positive outcome. Furthermore, goal based on population endpoints can be added. For NSCLC, a goal of $tcp>70\%$ and $ntcp<17.2\%$^17^, and for HCC, $tcp>90\%$ and $ntcp<25\%$ is added. Combining the reward and goal, the reward scheme for NSCLC and HCC is defined as following,

4 $$\begin{aligned} {r_{NSCLC}}= & {} {\left\{ \begin{array}{ll} r+2,&{}{\text {if}}\ tcp> 0.70\ \text {and}\ ntcp< 0.172,\\ r+1,&{}{\text {if}}\ tcp > 0.50\ \text {and}\ ntcp < 0.50,\\ {r,}&{}{\text {otherwise.}} \end{array}\right. } \end{aligned}$$

5 $$\begin{aligned} {r_{HCC}}= & {} {\left\{ \begin{array}{ll} r+2,&{}{\text {if}}\ tcp> 0.90\ \text {and}\ ntcp< 0.25,\\ r+1,&{}{\text {if}}\ tcp > 0.50\ \text {and}\ ntcp < 0.50,\\ {r,}&{}{\text {otherwise.}} \end{array}\right. } \end{aligned}$$

Note that goals might be unattainable for some patients.

**Figure 6:**
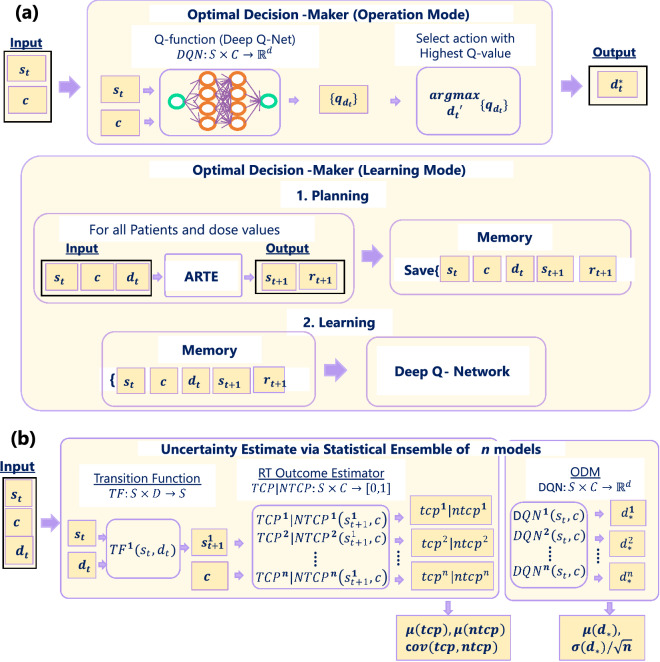
**(a)** Workflow of Optimal Decision-Maker (ODM). ODM is composed of a deep Q-network (DQN) and decision selector. Given a state $(s_t, c)$, DQN yields a q (quality) value for the range of adaptive dose. The selector greedily selects the dose with the highest q-value. The ODM is trained by following the model-based reinforcement learning paradigm. In the Planning Phase, the ODM saves next states $\{s_{t+1}\}$ and associated rewards $\{r_{t+1}\}$ for all patient’s state $\{(s_t,c)\}$ and the range of adaptive dose $\{d_t\}$. In the Learning phase, a double DQN algorithm is applied on the memory. **(b)** Model Uncertainty via Statistical Ensemble. We trained n identical models. The mean and covariance (or standard deviation) of the output distribution captures the model output and model uncertainty. For RTOE’s probability outputs, we present the uncertainty estimates as the covariance and for ODM dose recommendations, we present the uncertainty estimates as the standard error of mean.

### Optimal decision maker (ODM)

We utilized a deep reinforcement learning algorithm for training the ODM. ODM is composed of a Q (quality) function, $DQN:S\times C \rightarrow \mathbb {R}^d$, and a selector. Given a patient’s state $s_t$, deep Q-Net generates a set of q-values, $Q\subset \mathbb {R}^d$, for a range of dose. Q-Net maps k-dimensional state space to d-dimensional action (dose-decision) space. During operation mode, it simply follows greedy policy and selects the dose $d_t^{*}$ having the maximum q-value. For training, we have adopted a model-based RL paradigm that is divided into two phases: Planning and Learning, as shown in Fig. 6a. During Planning, an exhaustive search is carried out where all patient’s states $(s_t,c)$ and the range of adaptive dose $d_t$ are fed into the ARTE and the resulting states $s_{t+1}$, and rewards $r_{t+1}$ are saved into the Memory. During Learning, the DQN is trained via double DQN algorithms^31^ using the Memory as a one-step optimization problem.

### Uncertainty estimate via statistical ensemble

Model uncertainty is estimated using Statistical Ensembling. The statistical ensemble technique trains several identical models and finds averages and deviations of the prediction. This method estimates uncertainty purely based on the trained model. Additionally, this also helps with desensitizing ARCliDS to the noise associated with the stochastic optimization algorithm used by NNs. NNs utilize a large number of randomly initiated weights and as a result, learned weights are different from model to model^32^. ARCliDS presents the average prediction $\mu$ as an expected value, and the covariance *cov* as an uncertainty estimation as shown in Fig. 6b. For ODM, standard deviation $\sigma$ is used as the uncertainty estimate.

### Training and validation

A complete information on 67 NSCLC patients and 71 HCC patients were available for training and validation. A detailed description is provided in SM S7.1 and S8.1. We applied the 10-fold stratified shuffle for validation and hyper-parameter tuning of RTOE. The data set was split into 80–20% for training and for validation, respectively. We applied SMOTE and weighted loss function for correcting class imbalance. Basic hyper parameters—optimizer learning rate, number of neural network nodes, and training epoch—were tuned with the grid search algorithm. For further evaluation, we found Youden J-score and optimal thresholds from the training set and obtained the sensitivity and specificity of the validation set. Five RTOEs were then trained with the optimal hyper parameters on the whole data set. To increase the sample size for training ODM, we trained WGAN-GP on the available data. Trained WGAN-GP were then used to generate 10,000 synthetic patients. Five ODMs were trained in parallel by randomly sampling, with replacement, 4000 patients out of the 10,000 synthetic patients and feeding them to the 5 trained RTOEs. The trained ODM were then validated on the original datasets.

### Ethical approval statement

All methods were carried out in accordance with relevant NIH guidelines and regulations. All experimental protocols were approved by a ethics committee/IRB of University of Michigan. This work was carried out with the approval of ethics committee/IRB of Moffitt Cancer Center (MCC20750) and ethics committee/IRB of University of Michigan (Liver study:HUM00098022, Lung study: HUM00098202). Necessary informed consent was obtained from all subjects and/or their legal guardian(s). Identifiable data of subjects were not known to anyone outside the research group and were not used in any of this study, so cannot be used to identify the subjects.

## Supplementary Information

Supplementary Information.

## Data Availability

The data that support the findings of this study are available from the University of Michigan but restrictions apply to the availability of these data, which were used under data sharing protocol for the current study, and so are not publicly available. Data are however available from the corresponding author upon reasonable request and with permission of the University of Michigan. A formal written request regarding data sharing must be submitted to the Department of Radiation Oncology, University of Michigan.

## Code Availability

Software codes used in this study will be made available from the corresponding author on reasonable request.
