# Supplementary material for: A clinical decision support system for AI-assisted decision-making in response-adaptive radiotherapy (ARCliDS)
Source: Sci Rep. 2023 Mar 31;13:5279. doi: 10.1038/s41598-023-32032-6 (PMC10066294; doi:10.1038/s41598-023-32032-6)
Supplement: Supplementary file 1 — Supplementary Information. [file 41598_2023_32032_MOESM1_ESM.pdf]

Supplementary Materials - A Clinical Decision Support Software for AI-assisted Decision-Making in Response-Adaptive Radiotherapy (ARCliDS)

Dipesh Niraula[1], Wenbo Sun[2], Jionghua (Judy) Jin[3], Ivo D. Dinov[4], Kyle Cuneo[5], Jamalina Jamaluddin[6], Martha M. Matuszak[5,6], Yi Luo[1], Theodore S. Lawrence[5], Shruti Jolly[5], Randall K. Ten Haken[5], Issam El Naqa[1]

[1] Department of Machine Learning, H. Lee Moffitt Cancer Center & Research Institute, Tampa, FL 33612, USA.

[2] University of Michigan Transport Research Institute,
[3] Department of Industrial and Operations Engineering,
[4] Department of Health Behavior and Biological Sciences,
[5] Department of Radiation Oncology,
[6] Department of Nuclear Engineering and Radiological Sciences,

University of Michigan, Ann Arbor, MI 48109, USA.

Table of Contents

S1. Feature Selection and Graph Building via Markov Blanket Approach 3

S2. ARCliDS Training Sequence 4

S3. Transition Function for generalized equivalent uniform dose (gEUD) 6

S4. RT Outcome Estimator (RTOE) 8

S4.1 Generalized Logistic Function Guided Double GNN (GLoGD-GNN) for Monotonic TCP/NTCP 8

S4.2 RTOE Hyper Parameter (HP) Tuning and Validation 8

S5. Synthetic Patients via Generative Adversarial Network (GAN) 9

S6. Double Deep Q-Learning for Optimal Decision Maker (ODM) 10

Use Case 1: NSCLC 11

S7.1 Data Description 12

S7.2 GLoGD-GNN for Monotonic TCP/NTCP 17

S7.3 RTOE Hyper Parameter Tuning 19

S7.4 Synthetic Patient Generation via WGAN-GP 23

S7.5 ODM Decision Analysis 25

S7.5.1 DDQN trained on Single GNN RTOE - NSCLC 26

S7.5.2 DDQN trained on GLoGD-GNN RTOE- NSCLC 27

Use Case 2: HCC 28

S8.1 Data Description 29

S8.2 GLoGD-GNN for Monotonic TCP/NTCP 33

S8.3 RTOE Hyper Parameter Tuning 35

S8.4 Synthetic Patient Generation via WGAN-GP 39

S8.5 ODM Decision Analysis 41

S8.5.1 DDQN trained on Single GNN RTOE – HCC 42

S8.5.2 DDQN trained on GLoGD-GNN RTOE - HCC 43

S8.5.3 Experimentation with population-based reward goal for HCC 44

References 47

S1. Feature Selection and Graph Building via Markov Blanket Approach

In this work, we adopted the feature graph from Yi et al.'s work on multi-objective Bayesian networks for radiotherapy outcome prediction[1]. The steps of building an appropriate multi-objective Bayesian network (MO-BN) for joint prediction of TCP and NTCP include large-scale feature selection and network structure learning. The first step intends to identify important features from a high-dimensional dataset by exploring extended Markov blankets (MBs) of TCP and NTCP. An MB is an inner family found by constraint-based algorithms such as incremental association Markov blanket (IAMB) and Hiton approaches. The MB contains all variables carrying information about TCP and NTCP that cannot be obtained from any other variables. For each member in the MB of TCP and NTCP, a next-of-kin MB for this member can also be derived, which is combinedly known as extended MB.

The second step is to combine the important features from the extended MBs and search for the best stable MO-BN for joint prediction. After accommodating radiobiologically plausible relationships based on reported literature, Tabu Search is employed to generate a stable MO-BN structure from 300 randomly generated bootstrap samples. Bayesian Dirichlet equivalent (BDe) that provides an inherent penalty for model complexity is used as a scoring function to obtain the stable MO-BN. However, an initial stable network may not be the best model for joint TCP and NTCP prediction due to unimportant or redundant features in it. So, a leaf node is found out by increasing the arc threshold in the MO-BN generation. After removing the leaf node, a stable MO-BN can be generated again from Tabu Search. The process continues until the resulting MO-BN reaches its maximal prediction performance based on cross-validation. The network structure found for NSCLC and HCC are presented in Figures S5 and S18.

S2. ARCliDS Training Sequence

1. Train Artificial Radiotherapy Environment (ARTE) via supervised learning using patient's feature and dose plan as the input, and corresponding clinical outcomes as the label.
    a. Model Transition Function (TF) to take in mid-treatment feature (s_{eval}) and predict after-treatment feature (s_{adapt}) for the adaptation daily dose fractionation (d_{adapt})

    $$TF(s_{eval}, d_{adapt}) \rightarrow s_{adapt}$$

    More details are presented in Section S4.

    b. Train RT Outcome Estimators (RTOE) to estimate treatment outcome ($tcp,\ ntcp$) for the predicted s_{adapt} and covariate c as a binary classifier as shown in Figure S1.

    $$TCP(s_{adapt}, c_{eval}) \rightarrow tcp, \qquad NTCP(s_{adapt}, c_{eval}) \rightarrow ntcp$$

    More details are presented in Section S5.

    c. Design Reward Function that yields reward r which increases with increasing tcp and decreases with increasing $ntcp$. For instance:

    $$r = tcp(1 - ntcp)$$

2. Train Optimal Decision Maker (ODM) via deep reinforcement learning on a trained ARTE from step 1.
    a. Inputs $s_{eval}, c, and\ \ d_{adapt}$ to the ARTE and store $s_{adapt},\ tcp,\ ntcp$ and r for all GAN-generated synthetic patients

    $$\mathrm{ARTE}(\{s_{eval}, c, d_{adapt}\}) \rightarrow \{s_{adapt}, tcp, ntcp, r\}$$

    More details on GAN are presented in Section S6.

    b. Train ODM via Deep Q-learning as a one-step optimization process, i.e., one training episode for all patients representing the adaptive phase.

    $$\operatorname*{argmax}_{\theta} Q_{\theta}(s_{eval}, Q_{\theta'}(s_{eval}))$$

    More details are presented in Section S7.

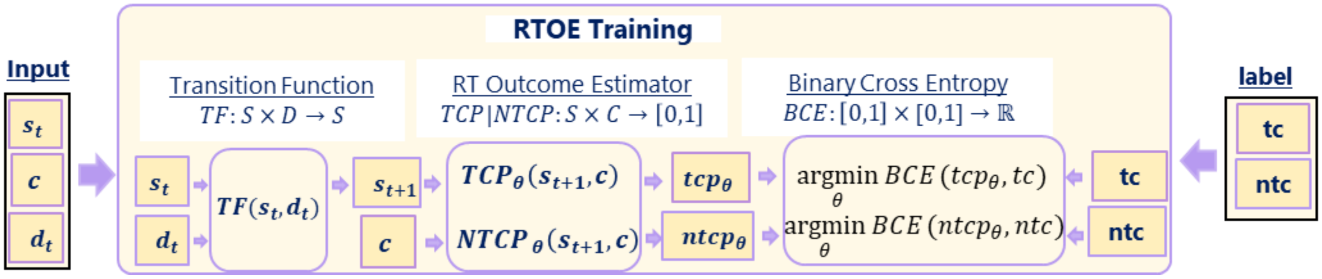

Figure S1: Training RTOE as a binary classification. Here θ represent tunable weights, which is learned by minimizing the binary cross entropy loss function. In the case of graph neural networks, the features are input as a directed graph $G(V, E)$, where the nodes, V, represents the features, $X \subset \mathbb{R}^{k \times 1}$ and edges, E, represents the inter-feature connections. Edges, E, are mathematically represented by adjacency matrix, $A \subset \mathbb{R}^{k \times k}$. During feedforward, the signal propagation is multiplied by the adjacency matrix given by $H^i = \sigma(AH^{i-1}\theta^i)$ for zero bias, where σ is the activation function, θ_i, is the weight of the ith hidden layer, and H_{i-1} is the matrix containing i-1st layer embeddings. Notice that multiplying by A preserves the only important inter-feature connections and eliminates computational redundancies; each node embedding is computed only once in contrast to fully connected neural network. For our case, features X is a concatenation of dosimetric variable, s, and other multi-omics covariate, c. Note, each patient is represented by a graph in the feature space and the binary classification is performed in the sample space as a graph classification problem[2].

S3. Transition Function for generalized equivalent uniform dose (gEUD)

GEUD absorbed by a patient must increase monotonically with increasing daily dose fractionation based on known principles of radiation biology[3]. To enforce this monotonic relation, we use a function of the form,

$$\frac{g(N_i) - g(N_{i-1})}{N_i - N_{i-1}} \propto \begin{cases} d_i\left(1 + \frac{d_i}{\frac{\alpha}{\beta}}\right) & \text{for } d_i < D_T \\ D_T + \frac{D_T^2}{\frac{\alpha}{\beta}} + \left(1 + \frac{2D_T}{\frac{\alpha}{\beta}}\right)(d_i - D_T) & \text{for } d_i \geq D_T \end{cases} \quad (S1)$$

where, i is the ith daily dose fractionation, g stands for gEUD, N stands for number of daily dose fractions, d stands for amount of dose fractions, D_T stands for threshold dose related to the linear-quadratic-linear (LQL) model, and α/β ratio is a parameter that differentiates tissue type.

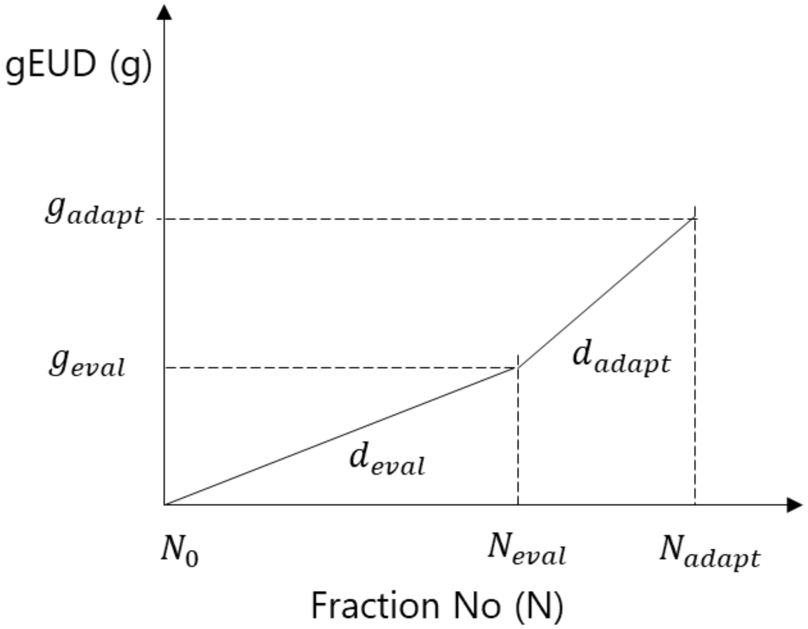

Figure S2: Transition Function for gEUD. Eval stands for the Evaluation Phase and adapt stands for the Adaptation Phase. Repeated from Figure 4 from the main text for clarity.

As shown in Figure S2**,** for our case, two relations arise from relation (S1), as follows:

$$\frac{g_{adapt} - g_{eval}}{N_{adapt} - N_{eval}} \propto \begin{cases} d_{adapt}\left(1 + \frac{d_{adapt}}{\frac{\alpha}{\beta}}\right) & \text{for } d_{adapt} < D_T \\ D_T + \frac{D_T^2}{\frac{\alpha}{\beta}} + \left(1 + \frac{2D_T}{\frac{\alpha}{\beta}}\right)(d_{adapt} - D_T) & \text{for } d_{adapt} \geq D_T \end{cases} , (S2)$$

And,

$$\frac{g_{eval} - g_0}{N_{eval} - N_0} \propto \begin{cases} d_{eval}\left(1 + \frac{d_{eval}}{\frac{\alpha}{\beta}}\right) & \text{for } d_{eval} < D_T \\ D_T + \frac{D_T^2}{\frac{\alpha}{\beta}} + \left(1 + \frac{2D_T}{\frac{\alpha}{\beta}}\right)(d_{eval} - D_T) & \text{for } d_{eval} \geq D_T \end{cases} . \quad (S3)$$

where, g stands for gEUD, N for nth daily dose fractions, d for dose fractions, D_T for threshold doses, and α/β ratio is a tissue-specific parameter. The subscript 0, eval, and adapt of N and g corresponds to pre-, mid-, and after-treatment, respectively while d_{eval} and d_{adapt} corresponds to applied daily dose fractionations during the evaluation phase and adaptive phase, respectively.

Furthermore, four scenarios can arise. The four different gEUD transition functions can be derived by taking the ratios of relations (S2) and (S3) as presented in Table S1.

Table S1: Transition Function for final gEUD (i.e., g_{adapt})

$\boldsymbol{g_{adapt}}$=	$d_{adpat} < D_T$	$d_{adapt} \geq D_T$
$d_{eval} < D_T$	$g_{eval}\left(1 + \left(\frac{n_{adapt}}{n_{eval}}\right)\left(\frac{d_{adapt}}{d_{eval}}\right)\left(\frac{d_{adapt} + \frac{\alpha}{\beta}}{d_{eval} + \frac{\alpha}{\beta}}\right)\right)$	$g_{eval}\left(1 + \left(\frac{n_{adapt}}{n_{eval}}\right)\left(\frac{\gamma d_{adapt} - D_T^2}{d_{eval}\left(d_{eval} + \frac{\alpha}{\beta}\right)}\right)\right)$
$d_{eval} \geq D_T$	$g_{eval}\left(1 + \left(\frac{n_{adapt}}{n_{eval}}\right)\left(\frac{d_{adapt}\left(d_{adapt} + \frac{\alpha}{\beta}\right)}{\gamma d_{eval} - D_T^2}\right)\right)$	$g_{eval}\left(1 + \left(\frac{n_{adapt}}{n_{eval}}\right)\left(\frac{\gamma d_{adapt} - D_T^2}{\gamma d_{eval} - D_T^2}\right)\right)$
$n_{adapt} \equiv N_{adapt} - N_{eval}$, $n_{eval} \equiv N_{eval} - N_0$, $\gamma \equiv \frac{\alpha}{\beta} + 2D_T$, $g_0 = 0$, $N_0 = 0$		

Note: For traditional RT, only the first scenario is relevant. However, for SBRT, all four scenarios come into play.

S4. RT Outcome Estimator (RTOE)

S4.1 Generalized Logistic Function Guided Double GNN (GLoGD-GNN) for Monotonic TCP/NTCP

Likewise, both TCP and NTCP must increase monotonically with increasing radiation dose. Due to the patient heterogeneity related noise in the data, RTOE composed of a single GNN classifier could not adequately represent the monotonic relationship. Thus, we developed a guided dual GNN architecture named GLoGD-GNN, which comprises of two GNNs, μ_{GNN} and T_{GNN} that is fed into a generalized logistic function along with the g_{adapt}. GloGD-GNN can be summarized as follows,

$$tcp/ntcp = \frac{1}{1 + \exp\left(\frac{g_{adapt} - \mu_{GNN}(s_{adapt})}{T_{GNN}(s_{adapt})}\right)}$$

where, μ_{GNN} and T_{GNN} are GNN's with sigmoid output layer and takes in s_{adapt} as input. GEUD g_{adapt} is min-max normalized before feeding it to the logistic function. During training, the weights of the GNN's are updated alternately; when one is training, the other is kept frozen.

S4.2 RTOE Hyper Parameter (HP) Tuning and Validation

We performed a grid search for HP tuning. We used Adam optimizer for the optimization and Area Under the Receiver Operating Characteristic Curve (AUROCC) as the performance metric. We applied 10-fold stratified shuffle 80-20 split on the imbalanced dataset, where the data sets were stratified according to the binary outcome so that each test sets contained the same ratios of the outcome class. In addition, we randomly oversampled the minority class of each training split. Both training and validation dataset were batched and randomly sampled during model training.

For the Single GNN architecture, we searched the following HP space:
Optimizer learning rate = [0.0001, 0.0005, 0.001, 0.005],
Number of nodes = [256, 128, 64], Training Epoch = [50, 100, 200, 300]

For the GloGD GNN architecture, the HP search space was as follows:
Optimizer learning rate for mu GNN = [0.0001, 0.0005, 0.001],
Optimizer learning rate for T GNN = [0.0001, 0.0005, 0.001],
Number of nodes = [256, 128, 64], Training Epoch = [100, 200, 300, 400]

After selecting the best HP that corresponded to the best average test AUROCC score, we reran validation using the best HP to test the reproducibility. The best HP and all of the original datasets were then used to train five RTOE models. The results from validation on the NSCLC and HCC datasets are summarized in Figure S3.

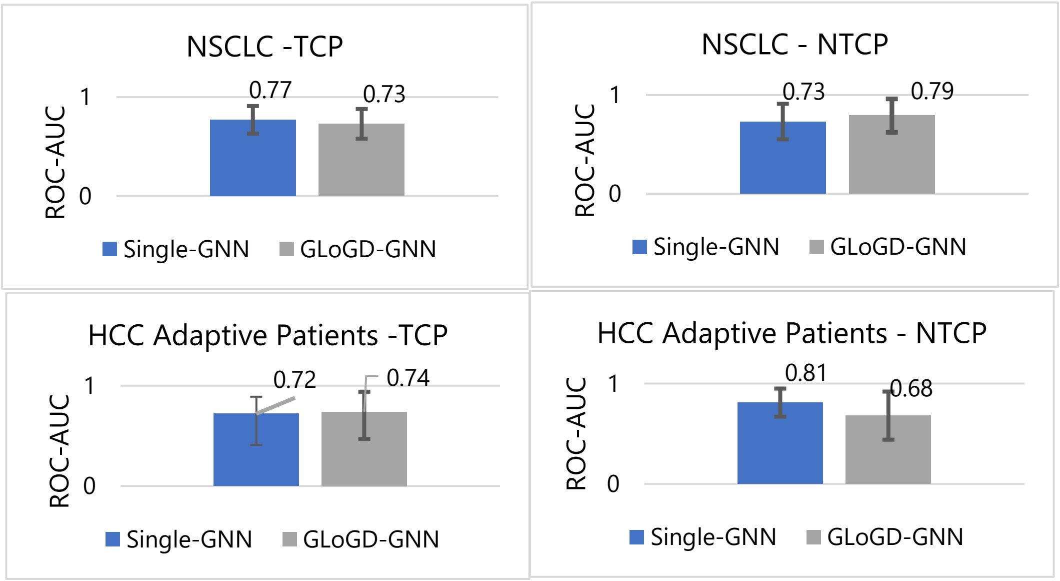

Figure S3: RTOE model performance for NSCLC and HCC data in Area Under the Receiver Operating Characteristic Curve (AUROCC). The mean AUROCC value is the area under the mean true positive rate curve, while the standard deviation is calculated from the AUROCC of 10 individual model output.

S5. Synthetic Patients via Generative Adversarial Network (GAN)

We applied a four-layer deep neural network with 256 nodes for both the discriminator and generator. We designed the generator to take in a 64-dimension normally distributed random numbers. We applied ADAM optimizer for the training with the learning rate of 1E-4, $\beta 1$ of 0.5, and $\beta 2$ of 0.9 and trained the GAN for 500 epochs. To maintain the stability by keeping discriminator ahead, we trained the discriminator 5 times for generator's every training epoch. As in the original work, we used a Gradient Penalty weight of 10 for our training. A comparison between the original and generated datasets are presented in Figures S13 and S26.

S6. Double Deep Q-Learning for Optimal Decision Maker (ODM)

ODM was trained using Double deep Q-learning (DDQN) algorithm. DDQN uses two neural networks, namely policy Q-net and target Q-net. This approach helps in correcting the overestimation of the q-values. We used a 5 layer deep 256 node wide architecture. For training parameters, batch size was set to 128, gamma (discount factor) to 0.8, Polyak factor to 0.99 (used for updating the target net's parameter) and Adam optimizer's learning rate to 0.0005.

For robustness, a planning and learning scheme were applied. Since in clinical setting, the physicians only get to select one action, we treated the problem as a one-step optimization problem, i.e., all episodes were terminated after one step. So, we performed an exhaustive search (i.e., explored all actions), before training the q-network. Note that this results in a greedy algorithm.

In the planning phase, 4000 out of 10,000 synthetic patients were randomly selected and their next states for all possible actions were exhaustively found using ARTE and stored. Additionally, the reward values and binary information on where they met the goal outcome were also stored. Then the DDQN was trained for 300 epochs. Huber loss was used as the loss function for updating the policy Q-net and Polyak update us used for updating the target Q-net.

The update rule for double deep Q-net learning is as follows,

$$Y_t \leftarrow R_{t+1} + \gamma Q_t\left(S_{t+1}, argmax_d Q_p(S_{t+1}, d; w_t), w_t^-\right).$$

Here, Y_t is the target value, γ is the discount factor, w_t is the weights of policy net Q_p, and w_t^- is the weights of the target network Q_t. The policy net is updated with the target value every step via stochastic gradient descent. However, the target net is kept almost fixed, only updating in small increment by copying parameters from target net via Polyak averaging, i.e.

$$w_{t+1}^- \leftarrow \alpha w_t^- + (1-\alpha) w_t$$

where α is the polyak factor.

For quantifying model uncertainty (or decision confidence), we trained 5 identical models.

Use Case 1: NSCLC

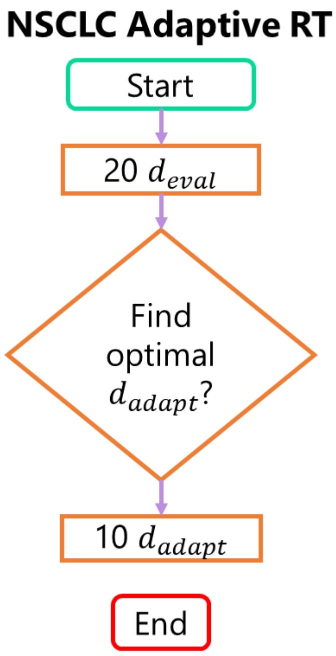

Figure S4: Clinical decision-making in NSCLC KBR-ART. In the evaluation phase, patients received 20 daily doses of preplanned fractions. After that the patients are evaluated, and an adaptive dose plan is designed and administered in 10 daily doses.

S7.1 Data Description

Dense multi-omics information on 117 patients were available. Out of that, 67 patients had complete information.

Table S2: NSCLC Patient Characteristics

Variable	Category	Patient Count (n =67)
Sex	Male	51
	Female	16
Age	Median (Q1-Q3)	66 (59-73)
	Range	55 – 85
Stage	I	4
	II	6
	III	57
Smoking	Yes	65
	No	6
COPD	Yes	28
	No	39
CVD	Yes	22
	No	45
Hypertension	Yes	41
	No	26
Histology	Adenocarcinoma	15
	Squamous Cell Carcinoma	27
	Large Cell	0
	Poorly Differentiated	25
Chemo	Yes	57
	No	10
Outcome	LC = 0	20
	LC = 1	47
	RP2 = 0	51
	RP1 = 0	16

COPD: Chronic obstructive pulmonary disease
CVD: Cardiovascular Diseases
LC: local control
RP2: Radiation induced pneumonitis of grade 2 or higher

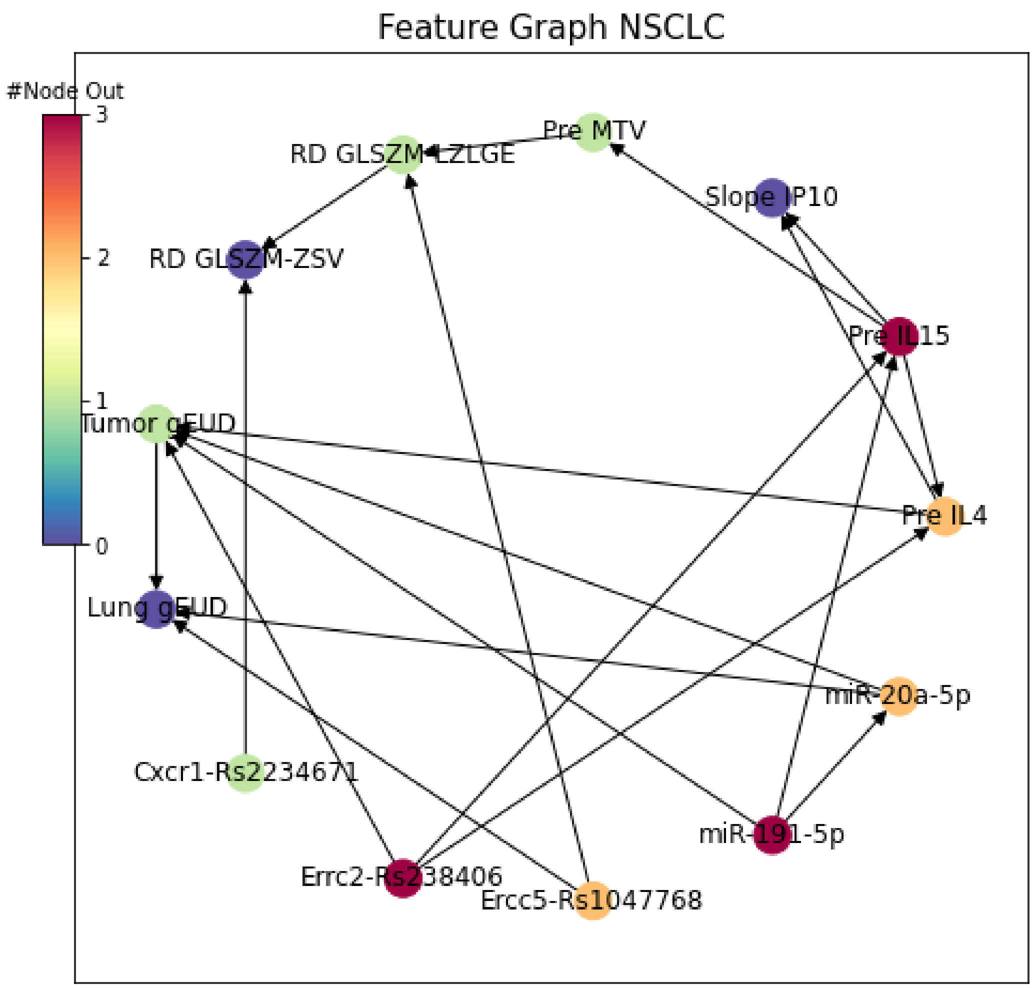

Figure S5: Directed graph showing the inter-relation between the NSCLC patient's features. The nodes, which represent features, are color coded with the number of outgoing relationships. Pre stands for pre-treatment observation, RD and slope stands for relative difference and change in feature value between pretreatment and mid-treatment observation, respectively.

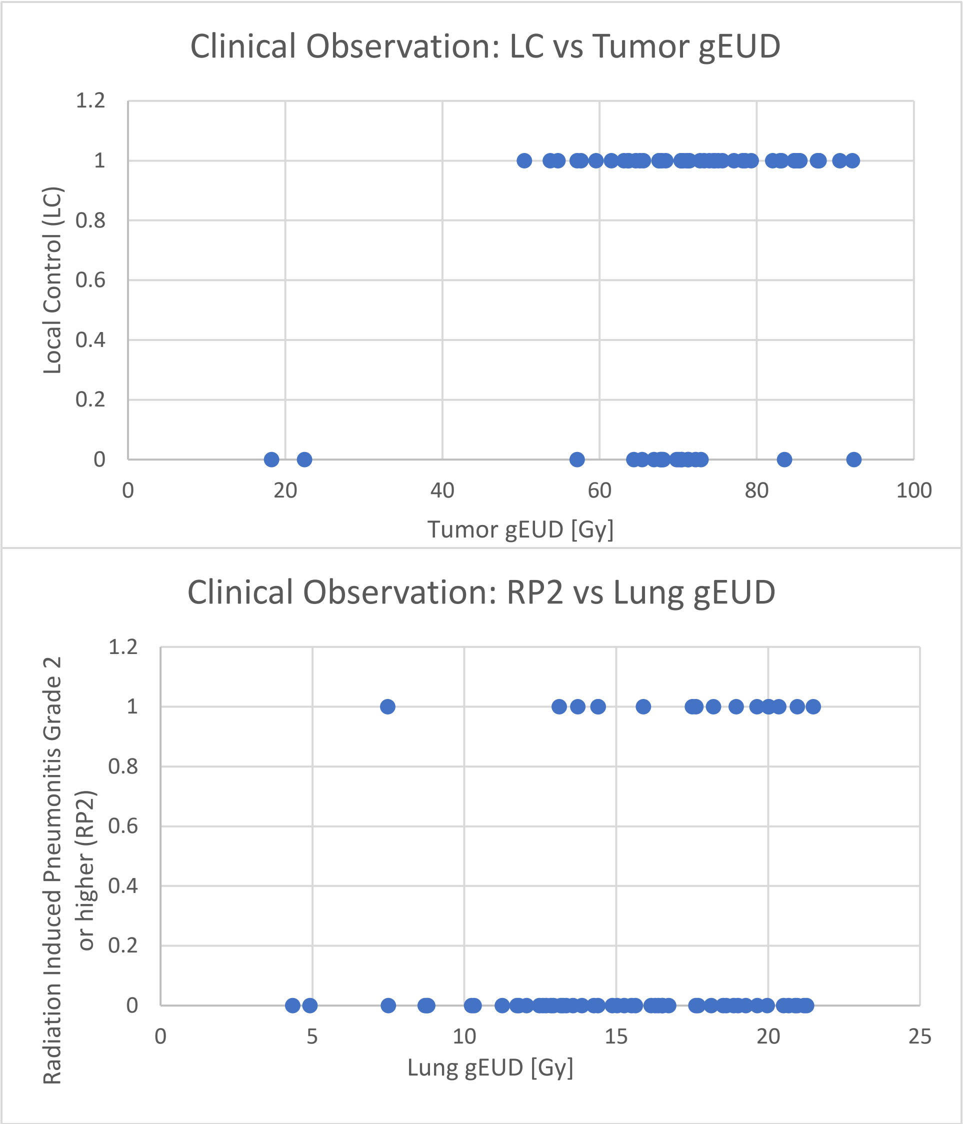

Figure S6: Plots showing NSCLC population tumor gEUD vs observed local control and lung gEUD vs observed radiation induced pneumonitis of grade 2 or higher. This plot captures inter-patient heterogeneity which shows patient's diverse treatment response.

Table S3: NSCLC Patients' feature description.

Patient Variable	Biological/Clinical Characteristics
**Cytokines/Signaling molecule**	
**IL4**	Interleukin 4 is a Th2 cytokines: (i) Regulates antibody production, hematopoiesis, and inflammation. (ii) Promotes the differentiation of naïve helper T cells into Th2 cells. (iii) Decreases the production of Th1 cells.[4–6]
**IL15**	Interleukin 15 is a Th2 cytokines: (i) Induces activation and cytotoxicity of NK (natural killer) cells. (ii) Activates macrophages. (iii) Promotes proliferation and survival of T and B- lymphocytes and NK cells.[4–6]
**IP10**	IP10 (Interferon gamma-induced protein 10) is secreted in response to IFN-γ by various cells including monocytes, endothelial and fibroblasts. (i) Acts as chemoattractant for monocytes/macrophages, T cells, NK (natural killer) cells, and dendritic cells. (ii) Promotes T cell adhesion to endothelial cells. (iii) Antitumor activity (iv) Inhibition of bone marrow colony formation (v) Angiogenesis.[4–6]
**CD4 T helper cells** are lymphocytes that strongly modulate the response of the immune system against cancer cells proliferation and tumor growth. They are classified into Th1 and Th2 cells. Th1 and Th2 cells generate Th1 and Th2 immune response by releasing cytokines. *Th1 immune response is proinflammatory and Th2 immune response in anti-inflammatory*.<br>**IFN-γ:** Th1 cytokines: (i) Enhances the microbicidal function of macrophages. (ii) Promotes the differentiation of naïve helper T cells into Th1 cells. (iii) Activates polymorphonuclear leukocytes, cytotoxic T cells, and NK cells. [4]	
**Tumor PET Imaging features/ Radiomics**	
**MTV**	Metabolic tumor volume was delineated from PET imaging using a method combining the tumor/aorta ratio auto segmentation and CT anatomy-based manual editing.[7]
**GLSZM-LZLGE**	Radiomics features: the large zone low gray-level emphasis (LZLGE) feature of a gray-level size zone matrix (GLSZM) is defined as $\sum_{i=1}^{N_g}\sum_{j=1}^{L_z}\frac{j^2 p(i,j)}{i^2}$. refer to Appendix A5 of Carrier-Valliere's thesis [8] for the Notations.
**GLSZM-ZSV**	Radiomics features: the zone-size variance (ZSV) feature of a gray-level size zone matrix (GLSZM) is defined as $\frac{1}{N_g\times L_z}\sum_{N_g}^{i=1}\sum_{L_z}^{j=1}\left(jp(i,j)-\mu_j\right)^2$, refer to Appendix A5 of Carrier-Valliere's thesis [8] for the Notations.
**Dosimetry**	
**Tumor gEUD**	Generalized equivalent uniform dose (gEUD) of tumor converted from EQD2 (Equivalent Dose at standard 2 Gy per fraction) dose distribution: $gEUD = (\sum_i \nu_i \mathrm{eqd}_2^a)^{\frac{1}{a}}$ and $\mathrm{eqd2} = N_{frac}\times d\times\frac{d+\alpha/\beta}{2+\alpha/\beta}$ *where* α/β= 10Gy, is the radiation fractionation sensitivity of cell, a = -10 is an organ specific parameter, and ν is the fractional organ volume obtained from the 3D dose distribution [9]
**Lung gEUD**	Generalized equivalent uniform dose (gEUD) of lung converted from EQD2 (Equivalent Dose at standard 2 Gy per fraction) dose distribution: $gEUD = (\sum_i \nu_i \mathrm{eqd}_2^a)^{\frac{1}{a}}$ and $\mathrm{eqd2} = N_{frac}\times d\times\frac{d+\alpha/\beta}{2+\alpha/\beta}$ *where* α/β= 4Gy, and a =1 [9]
**Genetics**	
**Cxcr1-Rs2234671**	A SNP in the gene cxcr1, also known as Interleukin 8 receptor alpha (IL8RA), related to radiation induced toxicity in non-small cell lung cancer. [10]
**Ercc2-Rs238406**	A SNP in the gene ercc2 known to repair DNA excision and related to risk of lung cancer [11]

**Ercc5-Rs1047768**	A SNP in the gene ercc5 also known to repair DNA excision and related to lung cancer susceptibility [12]
**SNP:** Single nucleotide polymorphism is a substitution of single nucleotide that occurs at a specific position in the genome via mutation.	
**MicroRNA**	
**miR-191-5p**	miR-191 is abnormally expressed in many cancer-types which regulate vital cellular processes such as cell proliferation, differentiation, apoptosis, and migration by targeting important transcription factors, chromatin remodelers, and cell cycle associated gene. [13]
**miR-20a-5p**	miR-20a has been found to be associated with lung cancer. It is encoded by a gene located on chromosome 13q31. It is involved in several oncogenic processes like cellular proliferation, angiogenesis, and apoptosis.[14]
MicroRNAs are small non-coding RNAs that negatively regular target gene expression through mRNA degradation or translation inhibition.[15]	

S7.2 GLoGD-GNN for Monotonic TCP/NTCP

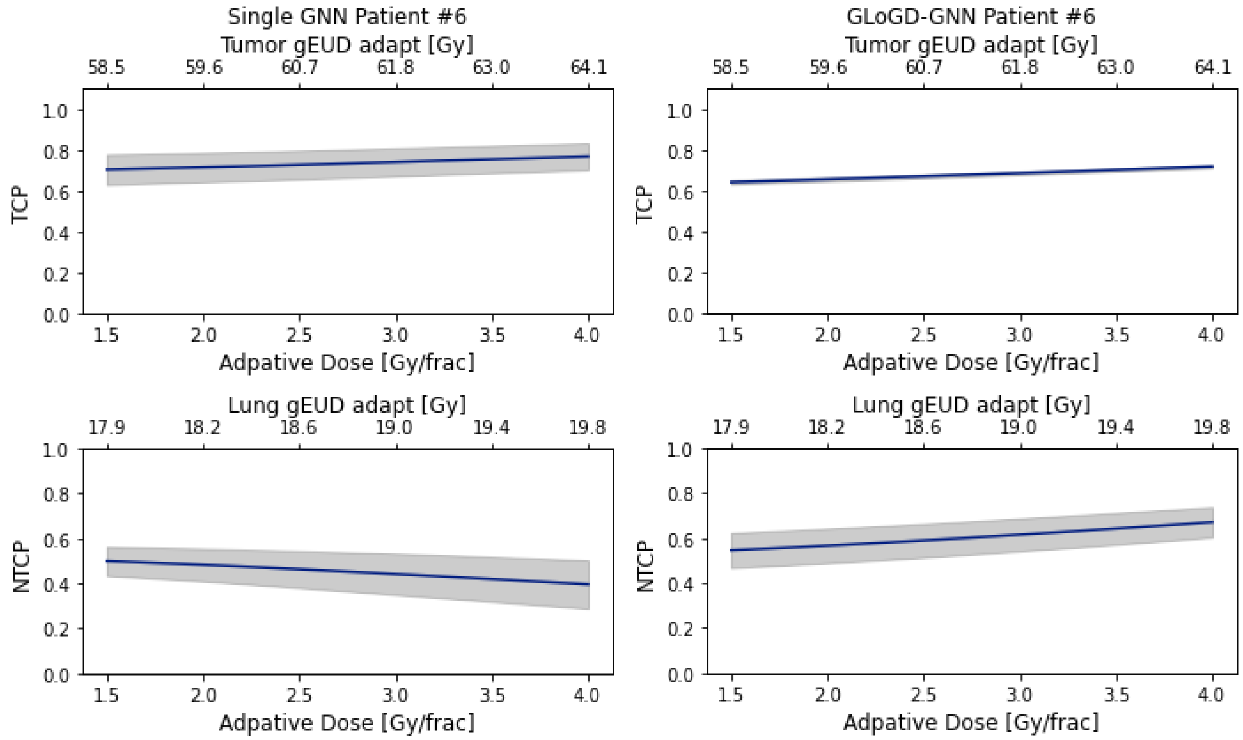

Figure S7: NSCLC outcomes estimate for adaptive dose ranging from 1.5 to 4.0 Gy/frac. The GLoGD-GNN correction successfully established a monotonic relationship for the NTCP. The model uncertainty is obtained from an ensemble of five RTOE model and presented as ± 1 standard deviation.

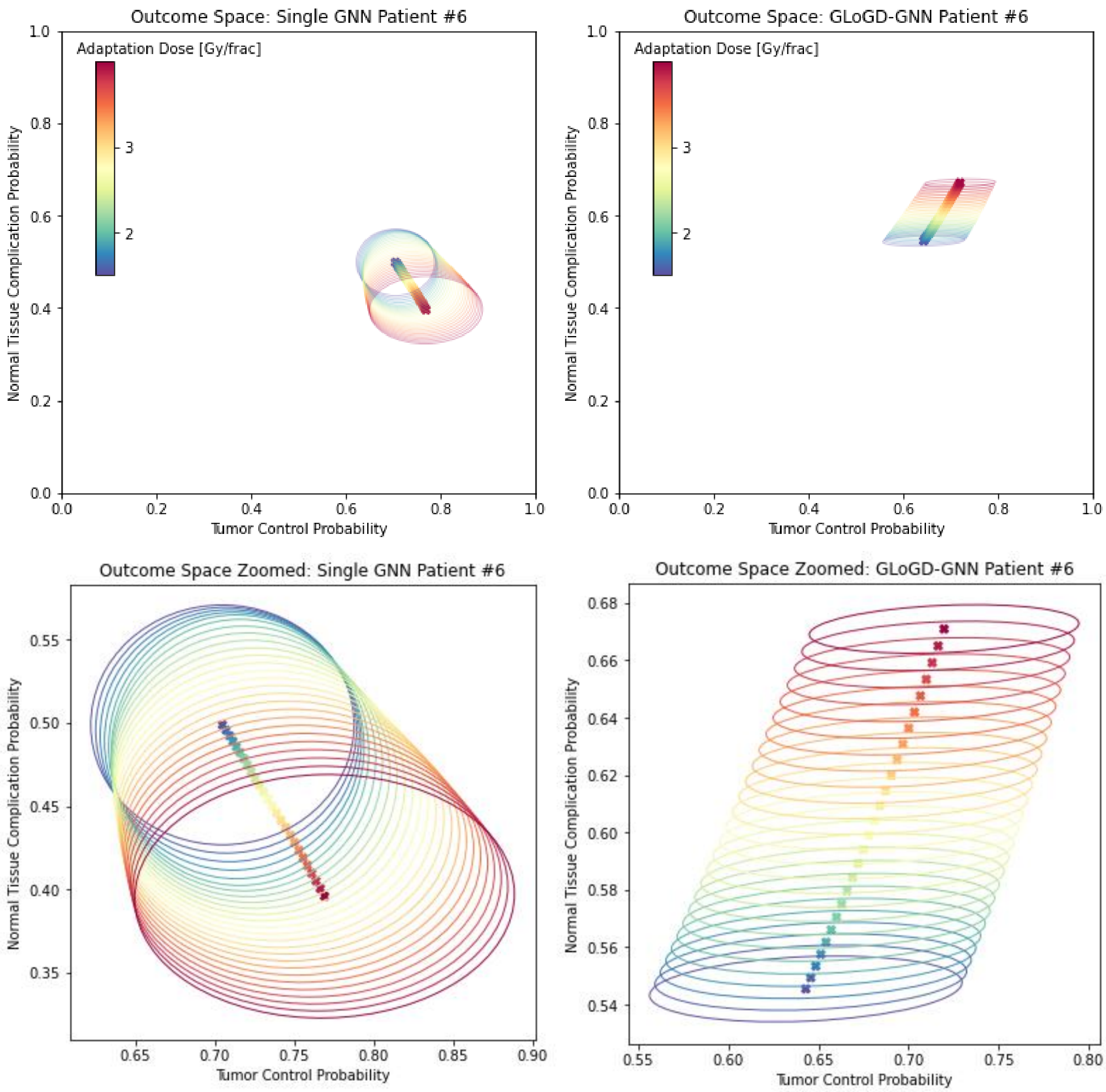

Figure S8: NSCLC outcomes estimate for adaptive dose ranging from 1.5 to 4.0 Gy/frac in the outcome space spanned by TCP and NTCP. Model uncertainty is obtained from an ensemble of five RTOE model and presented as an eclipse set by the Covariance matrix. In the left figure, NTCP is decreasing with increasing dose value. In the right figure, GLoGD-GNN correctly flipped the dose order i.e., NTCP is increasing with increasing dose value.

S7.3 RTOE Hyper Parameter Tuning

Tables S4-S7 lists the top 10 performing HP for the NSCLC dataset followed by receivers operating characteristics (ROC) for the best performing HP (marked in bold) shown in Figures S4-S12. To check for the reproducibility, ROC were generated by retraining the models with the best performing HP. The reported AUROC values are in the mean±stdev format, where the mean AUROCC value is the area under the mean true positive rate curve, while the standard deviation is calculated from the AUROCC of 10 individual model output.

Table S4: Top 10 HP for **NSCLC TCP (LC)** with **Single GNN**

HP #	*Opt lr*	*Node*	*Epoch*	*Training AUC*		*Avg Validation AUC*	
				Mean	*Stdev*	*Mean*	*Stdev*
**1**	**0.0001**	**128**	**50**	**0.85**	**0.03**	**0.79**	**0.10**
2	0.0001	64	200	0.88	0.02	0.76	0.12
3	0.0001	64	100	0.84	0.02	0.76	0.12
4	0.0001	128	100	0.88	0.02	0.76	0.10
5	0.0001	64	300	0.91	0.01	0.76	0.12
6	0.0001	256	50	0.88	0.03	0.76	0.11
7	0.0005	64	50	0.87	0.03	0.75	0.12
8	0.0001	64	50	0.82	0.02	0.75	0.14
9	0.0001	256	100	0.93	0.01	0.75	0.13
10	0.0005	128	50	0.93	0.02	0.74	0.13

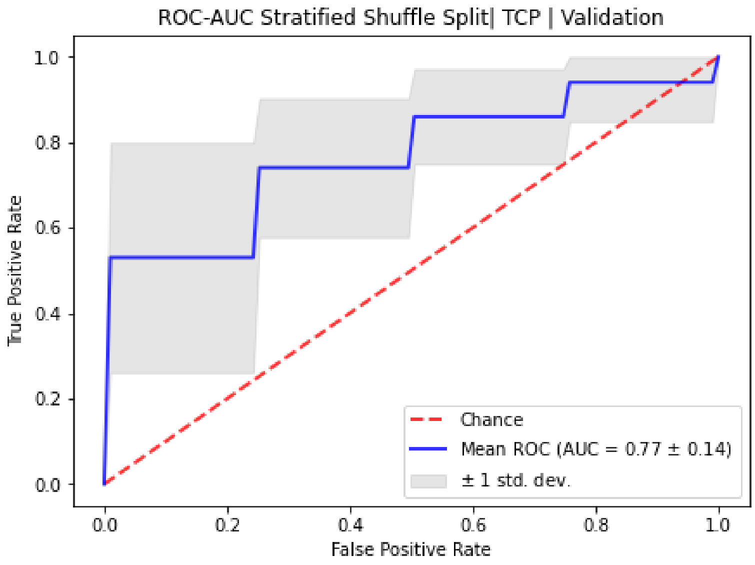

Figure S9: 10-fold stratified shuffle 80-20 split ROC for NSCLC RTOE of TCP modeled with Single GNN architecture.

Table S5: Top 10 HP for **NSCLC NTCP (RP2)** with **Single GNN**

HP #	*Opt lr*	*Node*	*Epoch*	*Training AUC*		*Avg Validation AUC*	
				Mean	*Stdev*	*Mean*	*Stdev*
**1**	**0.0001**	**64**	**50**	**0.77**	**0.04**	**0.75**	**0.13**
2	0.0001	128	50	0.83	0.04	0.72	0.17
3	0.0001	64	200	0.86	0.03	0.67	0.18
4	0.0001	64	100	0.82	0.03	0.67	0.18
5	0.0001	128	100	0.88	0.03	0.66	0.18
6	0.0001	256	50	0.87	0.02	0.64	0.18
7	0.0005	64	50	0.88	0.03	0.64	0.17
8	0.005	128	200	0.99	0.02	0.63	0.18
9	0.0001	64	300	0.91	0.03	0.62	0.16
10	0.001	64	50	0.93	0.02	0.62	0.18

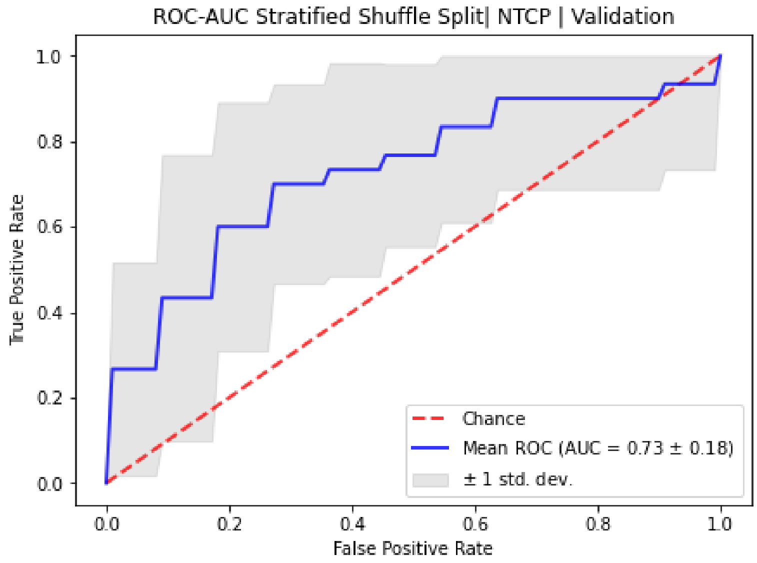

Figure S10: 10-fold stratified shuffle 80-20 split ROC for NSCLC RTOE of NTCP modeled with Single GNN architecture.

Table S6: Top 10 HP for **NSCLC TCP (LC)** with **GLoGD-GNN**

HP #	*Opt lr mu*	*Opt lr T*	*Node*	*Epoch*	*Training AUC*		*Avg Validation AUC*	
					Mean	*Stdev*	*Mean*	*Stdev*
**1**	**0.0001**	**0.0005**	**256**	**300**	**0.89**	**0.08**	**0.74**	**0.18**
2	0.0001	0.001	64	300	0.85	0.08	0.74	0.15
3	0.0001	0.001	64	200	0.84	0.06	0.73	0.15
4	0.0001	0.0005	64	100	0.83	0.03	0.72	0.15
5	0.0001	0.0001	64	100	0.83	0.01	0.72	0.16
6	0.0001	0.0005	128	400	0.88	0.08	0.72	0.16
7	0.0001	0.0005	64	200	0.87	0.05	0.72	0.13
8	0.0001	0.001	128	200	0.84	0.03	0.72	0.14
9	0.0001	0.0005	128	200	0.85	0.05	0.71	0.15
10	0.0001	0.0005	256	100	0.84	0.05	0.71	0.16

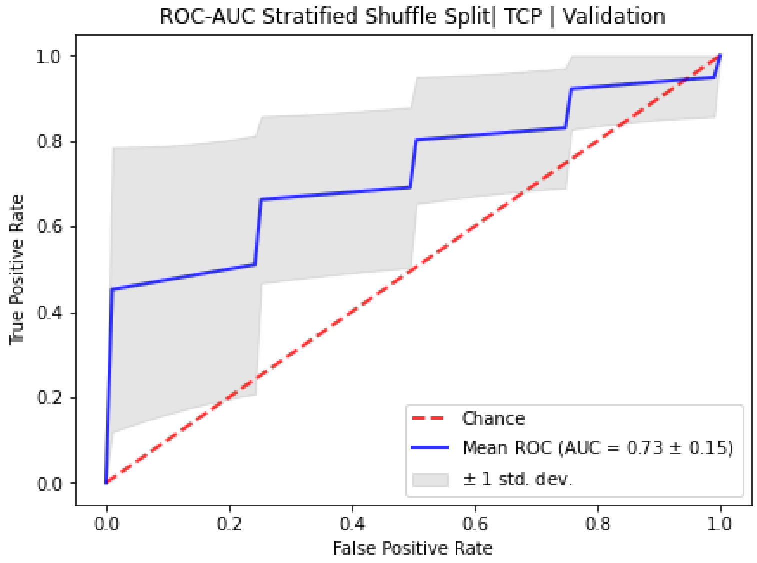

Figure S11: 10-fold stratified shuffle 80-20 split ROC for NSCLC RTOE of TCP modeled with GLoGD-GNN architecture.

Table S7. Top 10 HP for **NSCLC NTCP (RP2)** with **GLoGD-GNN**

HP #	*Opt lr mu*	*Opt lr T*	*Node*	*Epoch*	*Training AUC*		*Avg Validation AUC*	
					Mean	*Stdev*	*Mean*	*Stdev*
**1**	**0.0001**	**0.001**	**128**	**100**	**0.84**	**0.05**	**0.80**	**0.13**
2	0.0001	0.001	256	400	0.89	0.04	0.78	0.17
3	0.0001	0.001	256	300	0.85	0.11	0.76	0.19
4	0.0001	0.0001	256	400	0.88	0.07	0.76	0.19
5	0.0001	0.001	64	100	0.85	0.05	0.75	0.16
6	0.0001	0.0005	256	400	0.85	0.11	0.74	0.16
7	0.0001	0.0005	256	300	0.84	0.11	0.74	0.19
8	0.0001	0.0005	128	400	0.83	0.10	0.74	0.20
9	0.0001	0.001	256	100	0.84	0.08	0.74	0.16
10	0.0001	0.001	128	200	0.84	0.05	0.73	0.20

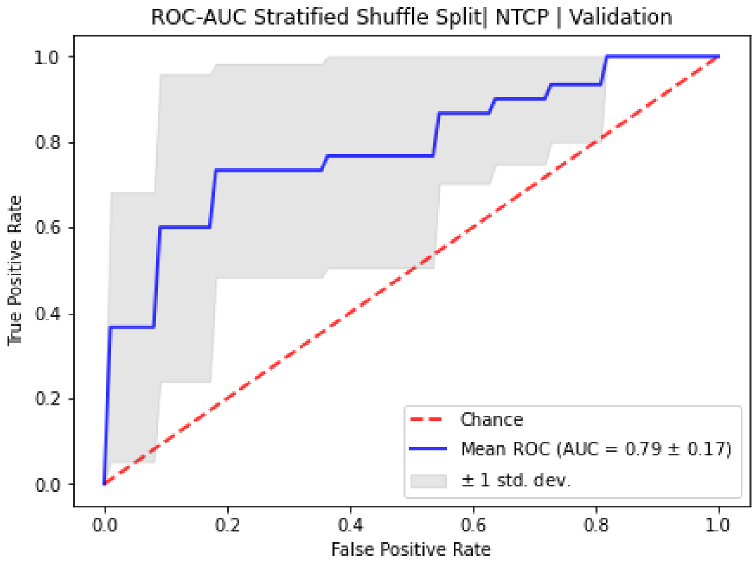

Figure S12: 10-fold stratified shuffle 80-20 split ROC for NSCLC RTOE of NTCP modeled with GLoGD-GNN architecture.

S7.4 Synthetic Patient Generation via WGAN-GP

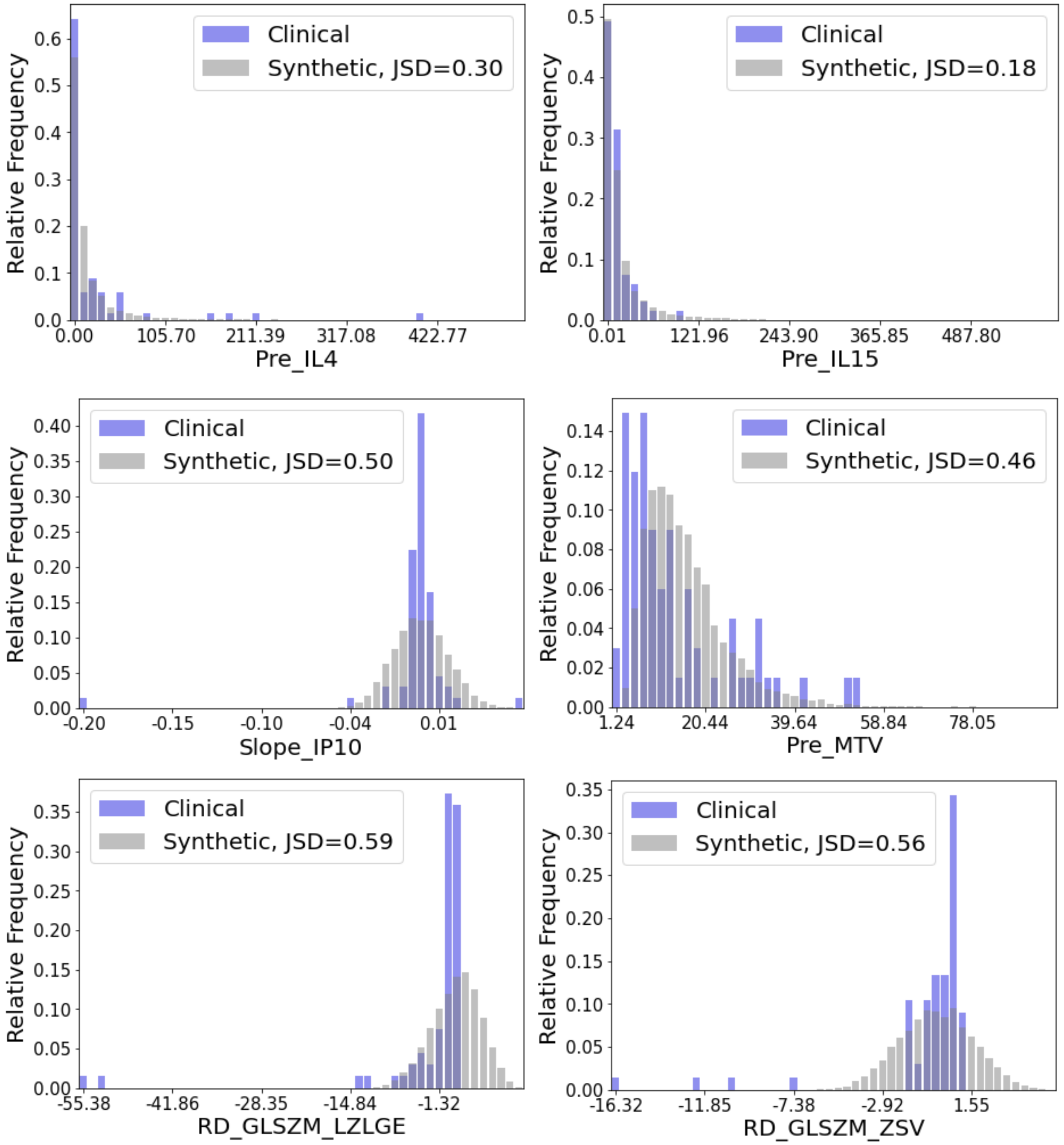

Figure S13: Distribution comparison of generated and original NSCLC dataset. Jensen Shannon Divergence metric between the distributions is provided for further insight on the differences.

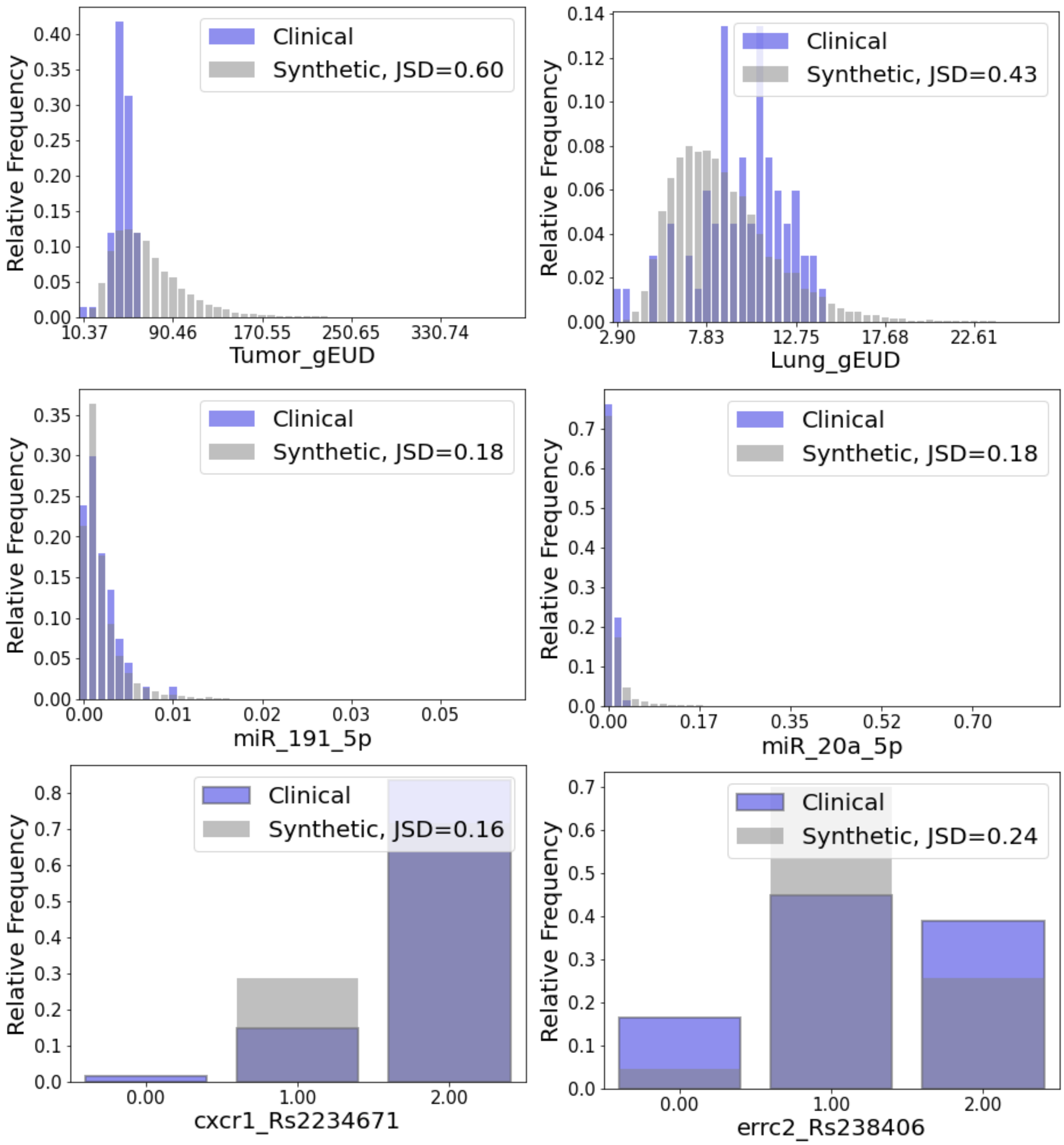

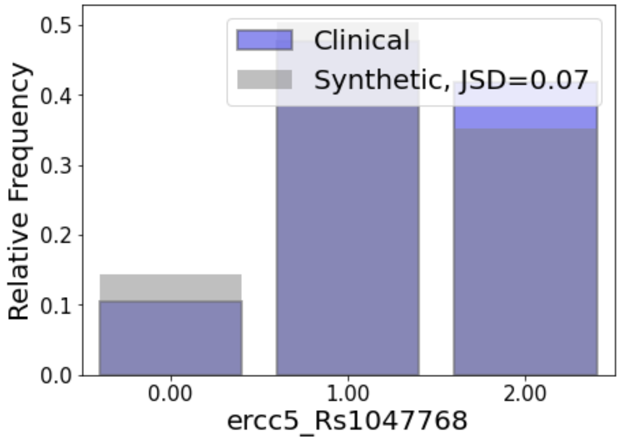

Figure S13 contd: Distribution comparison of generated and original NSCLC dataset. Jensen Shannon Divergence metric between the distributions is provided for further insight on the differences.

S7.5 ODM Decision Analysis

We trained the models on only the synthetic data and set aside the entire clinical dataset for testing. To further test the generalizability, each model was trained with 4000 out of 10,000 randomly chosen synthetic patients. After learning, the models were tested on the clinical data. We compared two model architectures as listed in Table S8 in terms of Root Mean Square Difference (RMSD) and Mean Absolute Difference (MAD) calculated with respect to the reported clinical decisions. For a level comparison, all architectures were trained under identical conditions.

Table S8: ODM Decision Analysis Results for NSCLC

Error [Gy/frac]	**Overall (n=67)**		**Positive Clinical Outcome (n=33 (49%))**		**Negative Clinical Outcome (n=34 (51%))**	
	RMSD	MAD	RMSD	MAD	RMSD	MAD
**Single GNN RTOE + DDQN ODM**	0.97 ± 0.12	0.85 ± 0.11	0.96 ± 0.11	0.85 ± 0.11	0.97 ± 0.12	0.84 ± 0.11
**GloGD GNN ROTE + DDQN ODM**	0.61 ± 0.03	0.51 ± 0.03	0.66 ± 0.02	0.58 ± 0.02	0.55 ± 0.05	0.43 ± 0.04

*Error ± SEM | Error between average recommendation and clinical decisions| ensemble of 5 models

Self-Evaluation [Count (%)]	**Overall (n=67)**			**Positive Clinical Outcome (n=33 (49%))**		**Negative Clinical Outcome (n=34 (51%))**	
	Good	Bad	Not Sure	Good	Not Sure	Good	Bad
**Single GNN RTOE + DDQN ODM**	26 (39%)	14 (21%)	27 (40%)	6 (18%)	27 (82%)	20 (59%)	14 (41%)
**GloGD GNN ROTE + DDQN ODM**	37 (55%)	9 (13%)	21 (31%)	12 (36%)	21 (64%)	25 (74%)	9 (26%)

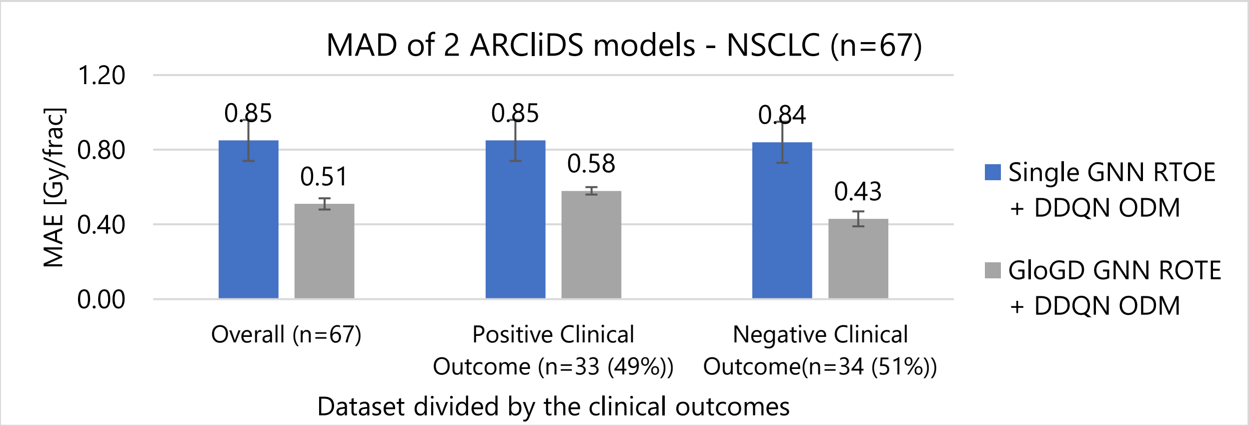

Figure S14: Mean Absolute Difference (MAD) of ARCliDS's two model architecture for NSCLC patients grouped together according to the outcomes.

S7.5.1 DDQN trained on Single GNN RTOE - NSCLC

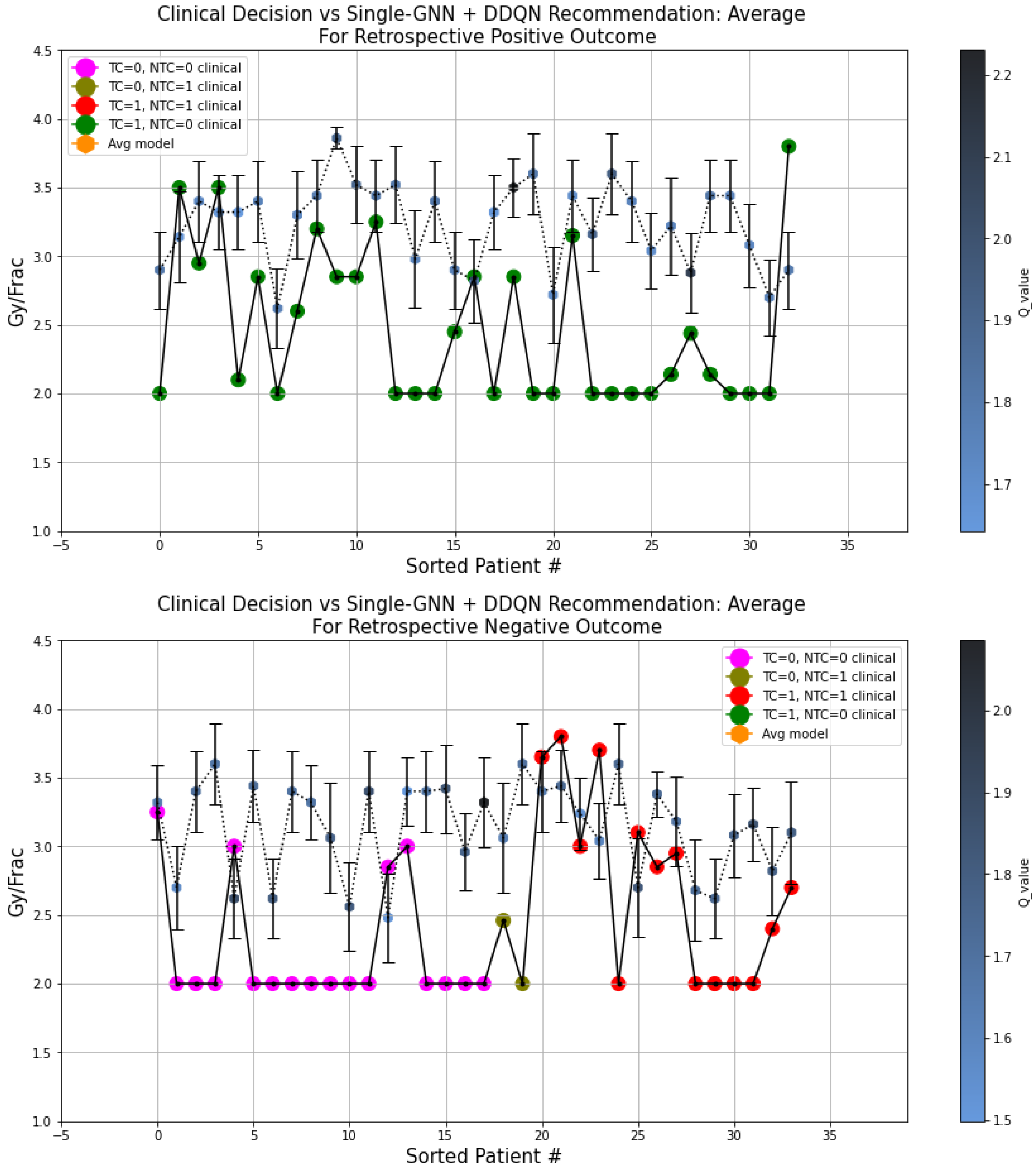

Figure S15: A visual comparison between the AI recommendation generated by the Single-GNN RTOE + DDQN ODM architecture and clinical decision for 2 groups of NSCLC patients divided according to the clinical outcomes. The clinical decisions are color coded with the outcomes and the ARCliDS recommendations are color coded with the respective q-value. Qualitatively, the q-value can be considered as the AI confidence in its recommendations.

S7.5.2 DDQN trained on GLoGD-GNN RTOE- NSCLC

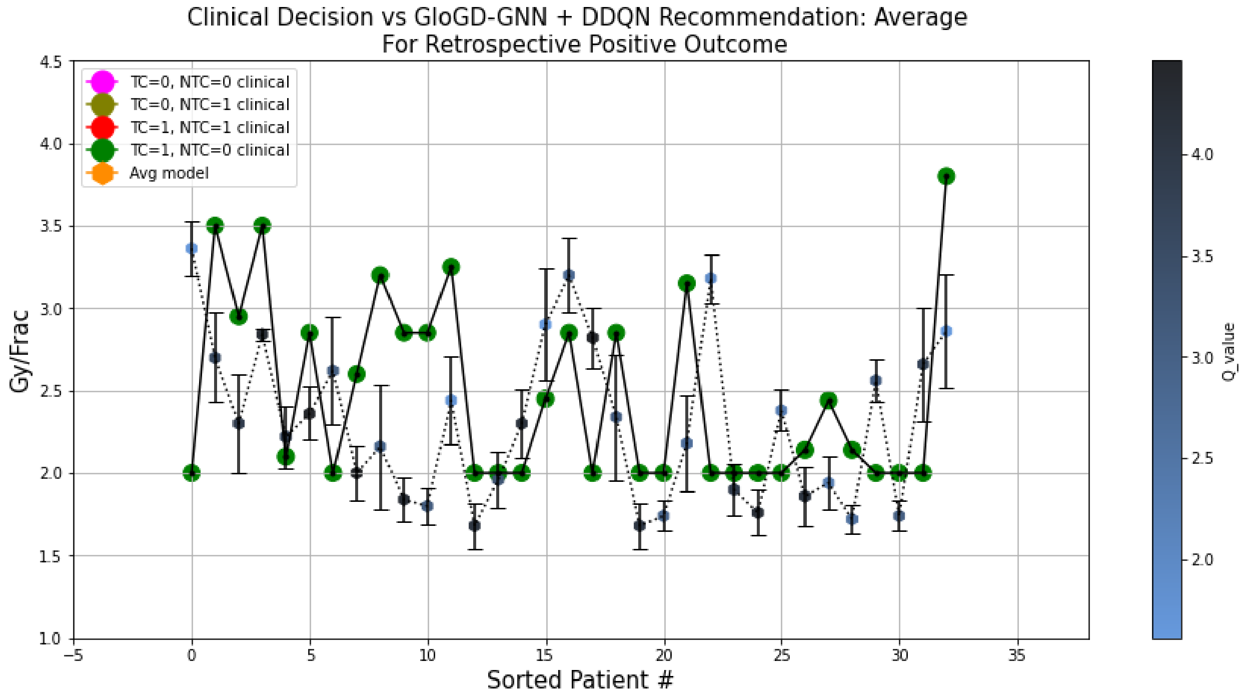

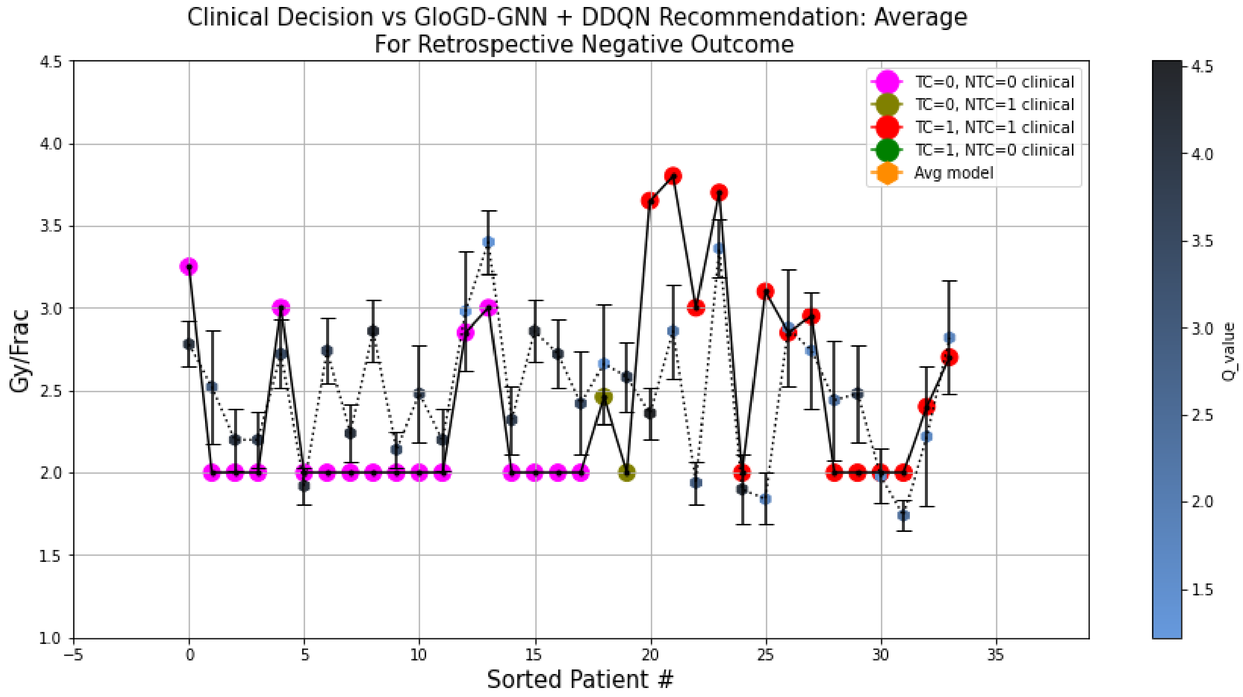

Figure S16: A visual comparison between the AI recommendation generated by the GLoGD-GNN RTOE + DDQN ODM architecture and clinical decision for 2 groups of NSCLC patients divided according to the clinical outcomes. The clinical decisions are color coded with the outcomes and the ARCliDS recommendations are color coded with the respective q-value. Qualitatively, the q-value can be considered as the AI confidence in its recommendations.

Use Case 2: HCC

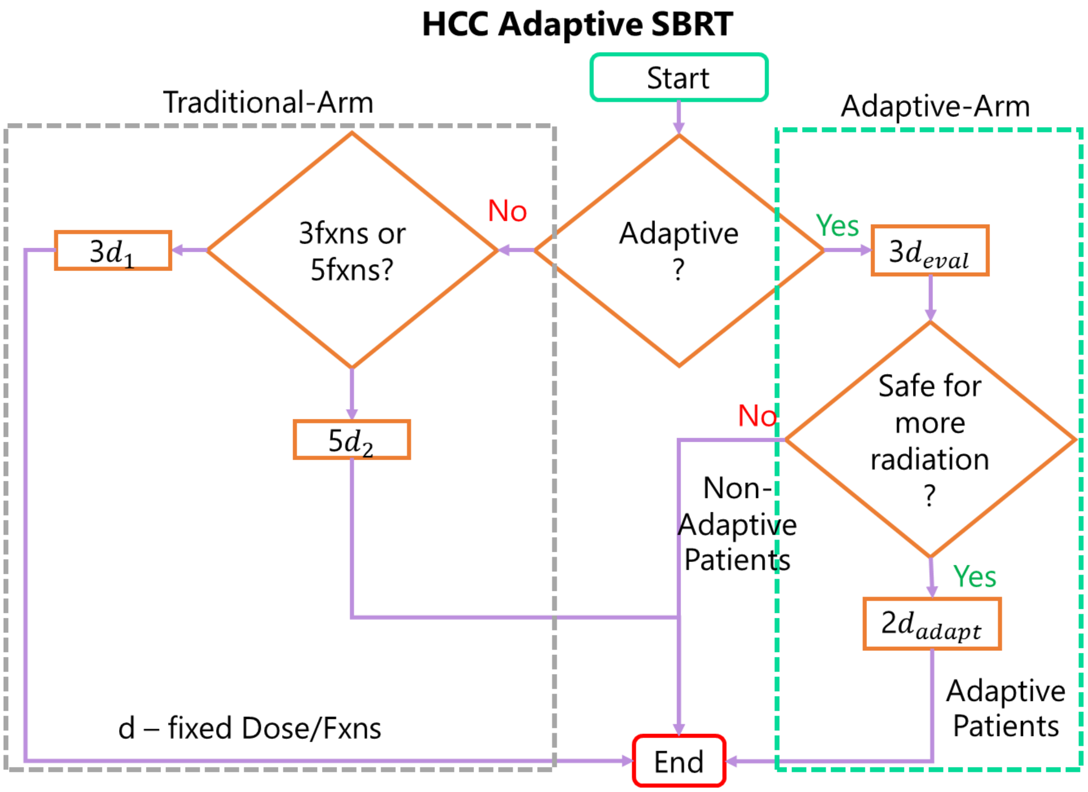

Figure S17: Decision-Making in HCC KBR-ASBRT. At first, patients are evaluated for the best course of treatment. Those that are not fit for adaptive SBRT receive traditional SBRT. The patients are further evaluated on whether they should receive the treatment in 3 or 5 fractions. The patients selected for Adaptive SBRT receive the first three fractions followed by a 1-month gap. After analyzing the trend in the multi-omics information, those deemed safe for further radiation recieves an adaptive dose (d_{adapt}) for 2 more fractions.

As shown in the Figure S17, decision making in the Adaptive Arm is a two-step process. First, the patient is given 3 daily dose fractions and evaluated if they are safe for more radiation. Second, for those that are safe, there is a question of what the optimal adaptive dose should be. The first decision can be decided by an RTOE, while the second decision by another RTOE and an ODM.

The Non-Adaptive RTOE is trained with input from non-adaptive patient's pre and mid treatment information and label from their outcome. The Adaptive RTOE is trained on the adaptive patient's dataset.

S8.1 Data Description

Information on 292 patients with 360 tumor sites were available. The greater number of tumor sites is due to multiple tumor or recurrence. Out of that, 81 patients with 104 tumor sites had dense multi-omics data available and 71 patients with 99 tumor sites had complete information.

Table S9: HCC Patient Characteristics

Variable	Category	Patient Count (n =71)
Sex	Male	56
	Female	15
Age	Median (Q1–Q3)	65 (59–75)
	Range	34–85
Pre-Treatment Cirrhosis	Yes	64
	No	7
Portal Vein Thrombosis	Yes	12
	No	59
Pre-Treatment Number of Active Lesions	Median (Q1-Q3)	1 (1–1)
	Range	1–4

Table S10: HCC Patient count classified by the treatment outcome.

Adaptive-Arm	Count HCC (n = 99)	Count HCC –Non-Adaptive (n = 36)	Count HCC –Adaptive (n = 64)
LC = 0	4	3	1
LC = 1	95	32	63
**Adaptive-Arm**	**Count HCC (n = 71)**	**Count HCC –Non-Adaptive (n = 30)**	**Count HCC –Adaptive (n = 41)**
LT = 0	54	20	34
LT = 1	17	10	7

LC: local control
LT: Liver Toxicity (≥ 2 points increase in Child Pugh Score during any point in the treatment.)
Total patients = 71, Non-adaptive Patient = 30, Adaptive Patients = 41,
For LC endpoints, patients with multiple tumor site were considered as different data points, Non-adaptive Datapoints = 36, Adaptive Datapoints = 64.
For LT endpoint, each patient was considered as one data point.

For the training of Adaptive RTOE, we added the 3 non-adaptive patients to the list of adaptive patients to increase the LC=0 count. To train RTOE for LC, the data from patients with multiple tumor sites were considered as different datapoints. Due to small sample size of non-adaptive patients, we considered only the second decision-making problem for the adaptive patients.

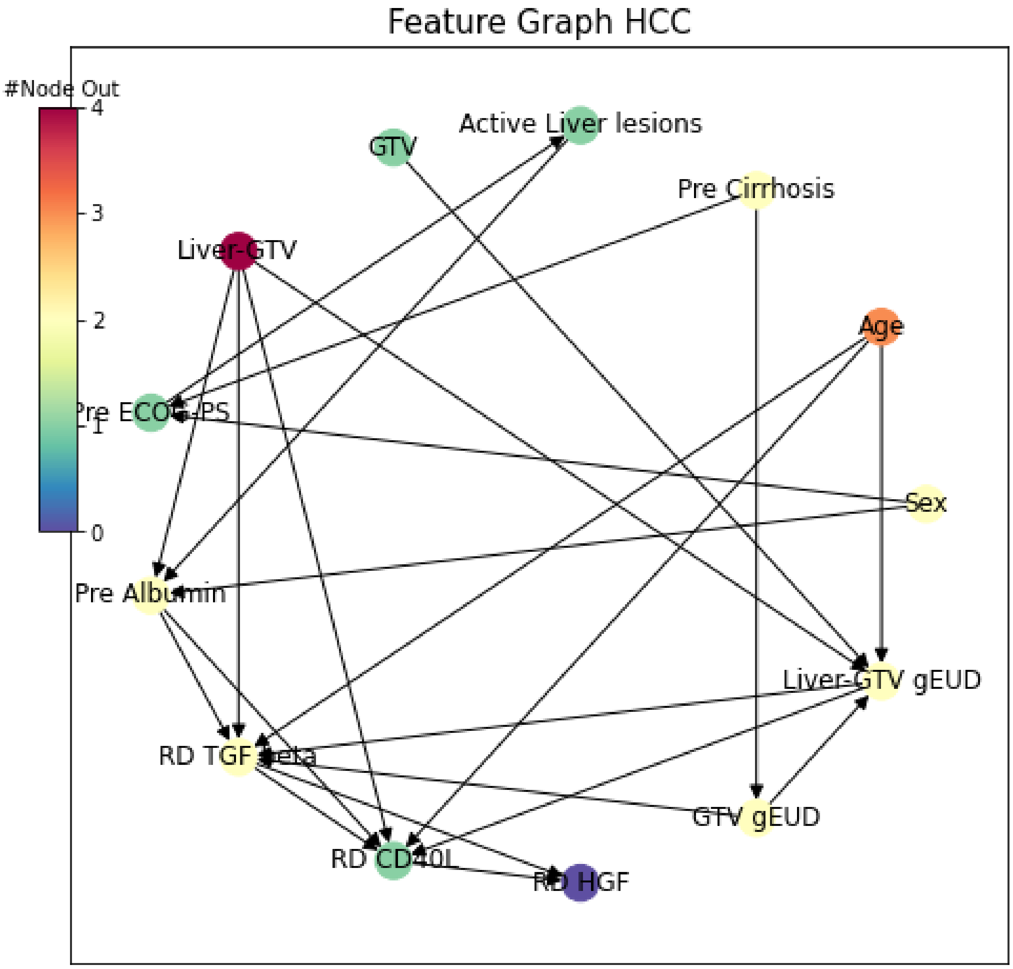

Figure S18: Directed graph showing the inter-relation between the HCC patient's features. The nodes, which represent features, are color coded with the number of outgoing relationships. Pre stands for pre-treatment observation, RD and slope stands for relative difference and change in feature value between pre-treatment and mid-treatment observation, respectively.

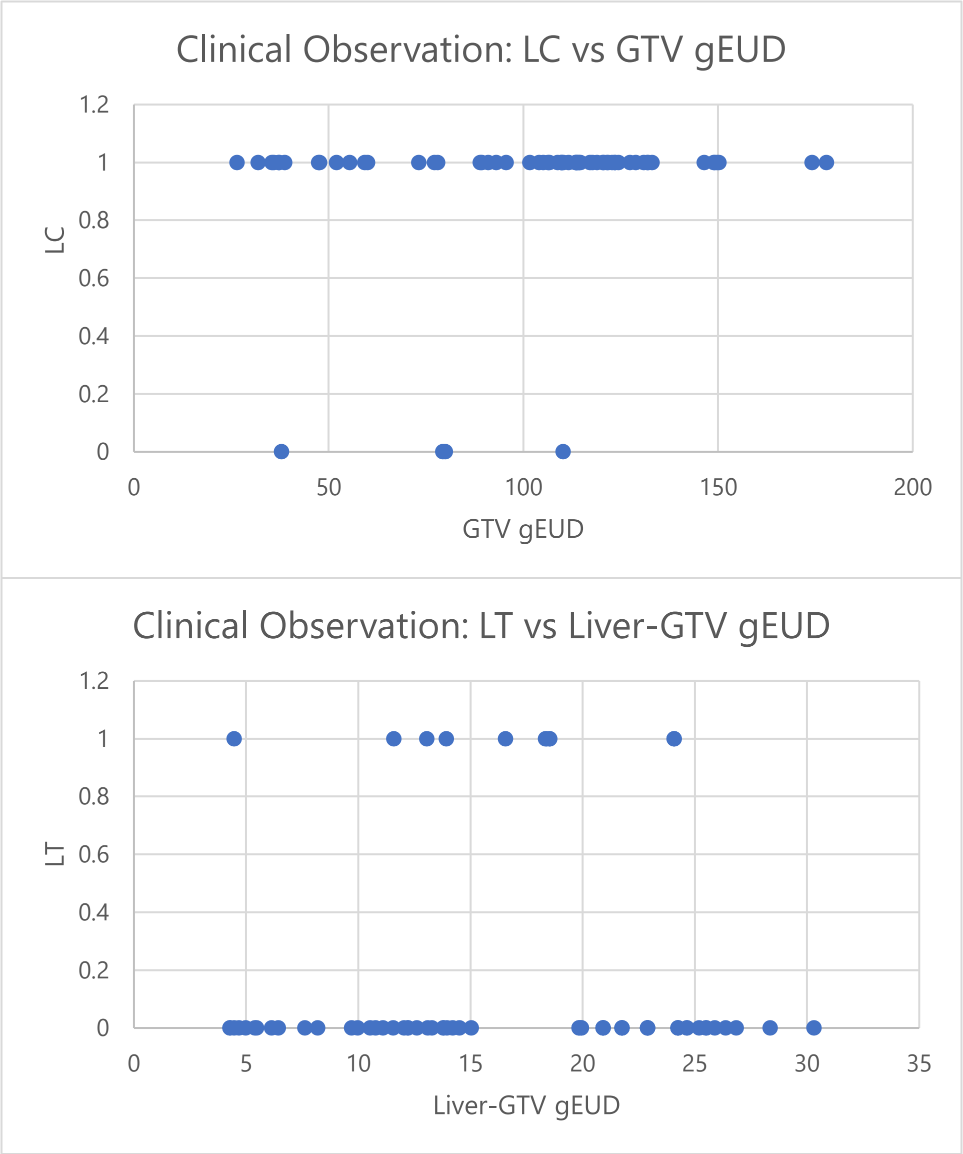

Figure S19: Plots showing HCC population GTV gEUD vs observed local control and Liver-GTV gEUD vs observed liver toxicity (LT). This plot captures inter-patient heterogeneity which shows patient's diverse treatment response. Note that, for population trend for LT is opposite to expected trend for an individual patient, i.e., patients with highest Liver-GTV gEUD is not showing toxicity.

Table S11: HCC Patients' feature description.

Patient Variable	Biological/Clinical Characteristics
**Clinical Factors**	
**Sex**	Biological male/female
**Age**	Pre-treatment Age
**Cirrhosis**	Late stage of scarring (fibrosis) of the liver
**ECOG-PS**	Eastern Cooperative Oncology Group Performance Status: ECOG-PS is a scale for assessing the level of function and capability of self-care. It ranges from integer 0 to 4 where PS0 indicates fully active patients while PS4 indicates patients that are completely unable for physical activity and self-care.
**Active Liver Lesions**	Abnormal clumps of cells in the liver
**Albumin**	A globular protein produced in the liver that circulates throughout human body via plasma. Its synthesis is stimulated by hormones such as insulin and inhibited by pro-inflammatory substance such as IL6 and TNF-α.[16]
**Tumor PET Imaging**	
**GTV**	Gross tumor volume is the volume of the actual tumor.
**Liver-GTV**	Liver volume minus GTV. This provides the volume of the normal liver tissue.
**Dosimetry**	
**GTV gEUD**	Generalized equivalent uniform dose (gEUD) of GTV converted from EQD2 (Equivalent Dose at standard 2 Gy per fraction) dose distribution using the Linear-Quadratic-Linear model. The model parameters used are α/β = 10 Gy, a=-20, and D_T=20 Gy.[9]
**Liver-GTV gEUD**	Generalized equivalent uniform dose (gEUD) of liver volume minus GTV converted from EQD2 (Equivalent Dose at standard 2 Gy per fraction) dose distribution using the Linear-Quadratic-Linear model. The model parameters used are α/β = 2.5 Gy, a=1, and D_T=5 Gy.[9]
**Cytokines/Signaling molecule**	
**TGF-$\boldsymbol{\beta}$**	Transforming growth factor beta is the prototype of TGF-β family that has diverse role in the control of cell proliferation and differentiation, wound healing and immune system, and pathology such as skeletal diseases, fibrosis, and cancer.[17]
**CD40L**	Cluster of Differentiation 40 receptor is a costimulatory molecule from the tumor necrosis factor receptor (TNF-R) family. CD40 binds with its ligand (CD40L) which is transiently expressed on T cells (immune cell) as well as other non-immune cells, under inflammatory conditions. This activates antigen presenting cells and other wide spectrum of molecular and cellular process. [18]
**HGF**	Hepatocyte growth factor is a pleiotropic cytokine required for development of several organs. It is produced after injury of the organ tissue and promotes tissue repair. HGF promotes tissue repair through inhibition of apoptosis of epithelial and endothelial cells, and by counteracting several pro-apoptotic and fibrosis factors such as TGF-β, IL-1β, IL8, TNF-α.[19,20]

S8.2 GLoGD-GNN for Monotonic TCP/NTCP

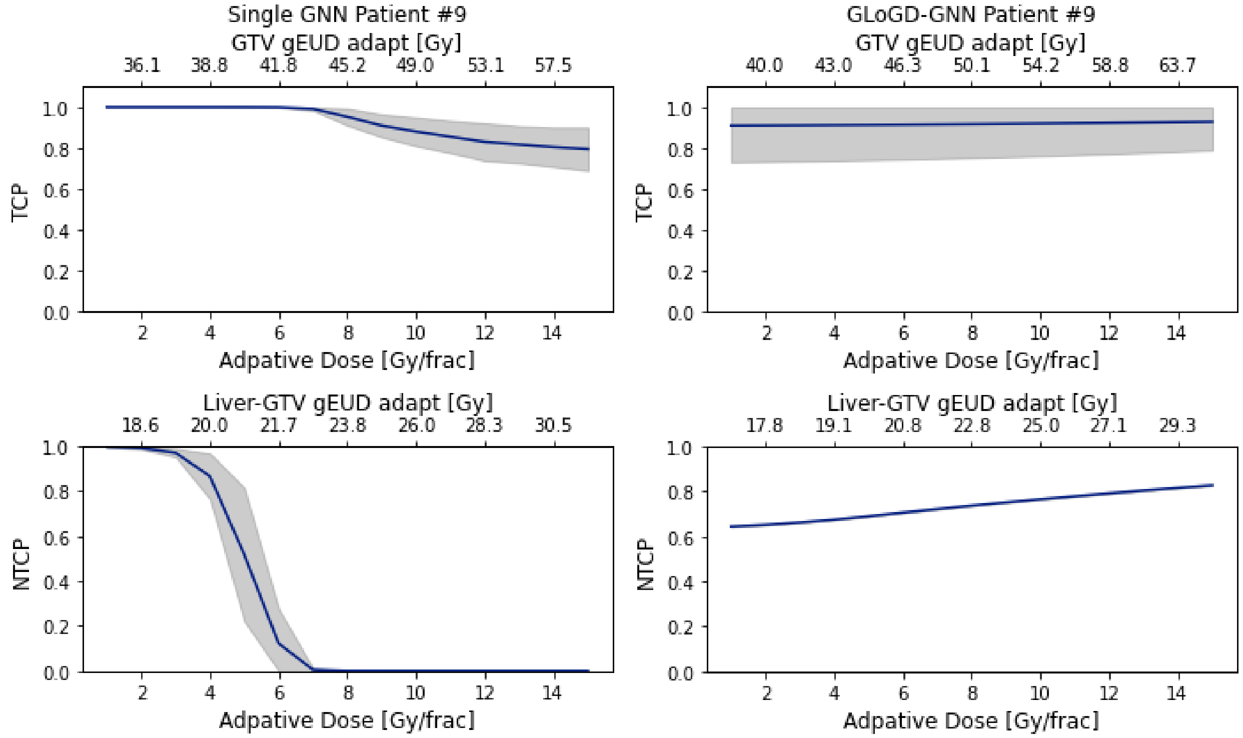

Figure S20: HCC outcomes estimate for adaptive dose ranging from 1 to 15 Gy/frac. The GLoGD-GNN correction successfully established a monotonic relationship for the NTCP. The model uncertainty is obtained from an ensemble of five RTOE model and presented as ± 1 standard deviation.

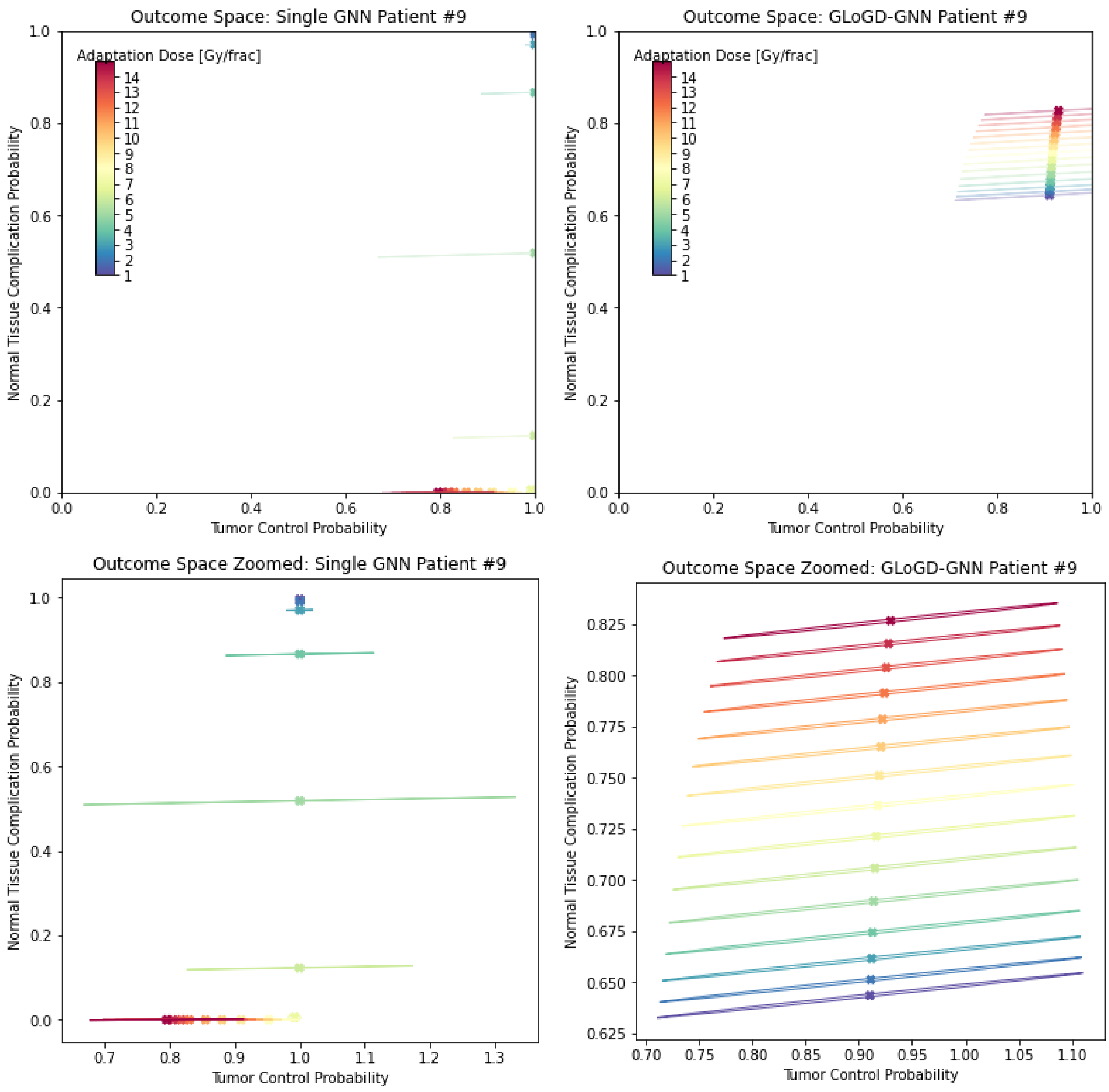

Figure S21: NSCLC outcomes estimate for adaptive dose ranging from 1 to 15 Gy/frac in the outcome space spanned by TCP and NTCP. Model uncertainty is obtained from an ensemble of five RTOE model and presented as an eclipse set by the Covariance matrix. In the left figure, NTCP is decreasing with increasing dose value. In the right figure, GLoGD-GNN correctly flipped the dose order i.e., NTCP is increasing with increasing dose value.

S8.3 RTOE Hyper Parameter Tuning

Tables S12-S15 lists the top 10 performing HP for the HCC dataset followed by receivers operating curves (ROC) for the best performing HP (in bold) shown in Figures S22-S25. To check for the reproducibility, ROCs were generated by retraining the models with the best performing HP. The reported AUROCC values are in the mean±stdev format, where the mean AUROCC value is the area under the mean true positive rate curve, while the standard deviation is calculated from the AUROCC of 10 individual model output.

Table S12: Top 10 HP for **HCC Adaptive TCP** with **single-GNN**

HP #	*Opt lr*	*Node*	*Epoch*	*Training AUC*		*Avg Validation AUC*	
				Mean	*Stdev*	*Mean*	*Stdev*
**1**	**0.005**	**64**	**200**	**0.99**	**0.00**	**0.76**	**0.31**
2	0.0001	64	100	0.99	0.00	0.74	0.31
3	0.001	256	200	0.99	0.00	0.73	0.30
4	0.001	128	300	0.99	0.00	0.73	0.32
5	0.005	64	300	0.99	0.00	0.72	0.30
6	0.001	256	100	0.99	0.00	0.72	0.33
7	0.001	256	300	0.99	0.00	0.72	0.30
8	0.001	256	50	0.99	0.00	0.72	0.33
9	0.005	128	200	0.99	0.00	0.72	0.34
10	0.0005	256	300	0.99	0.00	0.71	0.32

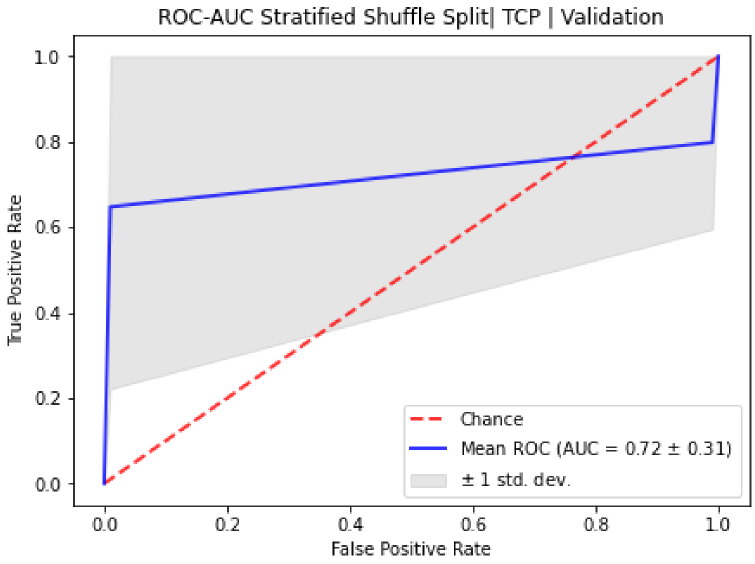

Figure S22: 10-fold stratified shuffle 80-20 split ROC for HCC Adaptive RTOE of TCP modeled with single-GNN architecture. Note: High Validation AUC deviation and relatively flatter AUC curve is due to severe class imbalance.

Table S13: Top 10 HP for **HCC Adaptive NTCP** with **single-GNN**

HP #	*Opt lr*	*Node*	*Epoch*	*Training AUC*		*Avg Validation AUC*	
				Mean	*Stdev*	*Mean*	*Stdev*
**1**	**0.005**	**128**	**100**	**0.99**	**0.02**	**0.81**	**0.13**
2	0.0001	128	300	0.99	0.00	0.80	0.12
3	0.001	256	50	0.99	0.00	0.80	0.12
4	0.001	256	300	0.99	0.00	0.80	0.14
5	0.0001	128	200	0.99	0.00	0.78	0.11
6	0.001	128	50	0.99	0.00	0.78	0.11
7	0.005	64	50	0.99	0.01	0.78	0.15
8	0.0005	256	100	0.99	0.00	0.78	0.12
9	0.001	256	200	0.99	0.00	0.78	0.13
10	0.005	64	100	0.99	0.00	0.78	0.17

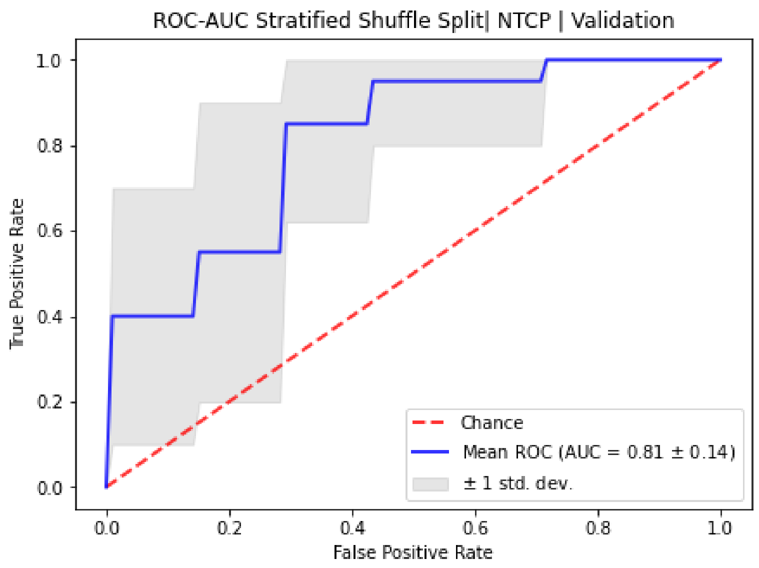

Figure S23: 10-fold stratified shuffle 80-20 split ROC for HCC Adaptive RTOE of NTCP modeled with Single GNN architecture.

Table S14: Top 10 HP for **HCC Adaptive TCP** with **GLoGD-GNN**

HP #	*Opt lr mu*	*Opt lr T*	*Node*	*Epoch*	*Training AUC*		*Avg Validation AUC*	
					Mean	*Stdev*	*Mean*	*Stdev*
**1**	**0.0005**	**0.0001**	**256**	**300**	**0.92**	**0.13**	**0.77**	**0.21**
2	0.0005	0.0005	256	100	0.87	0.14	0.76	0.19
3	0.0001	0.0005	256	200	0.89	0.20	0.75	0.21
4	0.0001	0.001	256	400	0.85	0.22	0.73	0.21
5	0.0001	0.001	128	100	0.91	0.15	0.72	0.20
6	0.0001	0.0005	128	300	0.76	0.24	0.72	0.21
7	0.0001	0.001	128	200	0.81	0.21	0.71	0.22
8	0.0001	0.0005	256	300	0.94	0.15	0.71	0.21
9	0.0005	0.0001	64	200	0.99	0.00	0.71	0.28
10	0.0001	0.001	128	400	0.97	0.06	0.69	0.26

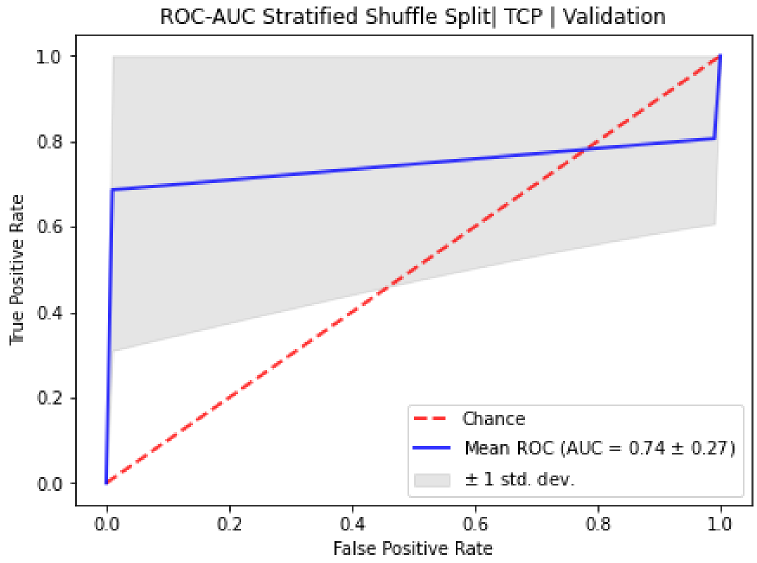

Figure S24: 10-fold stratified shuffle 80-20 split ROC for HCC Adaptive RTOE of TCP modeled with GLoGD-GNN architecture. Note: High Validation AUC deviation and relatively flatter AUC curve is due to severe class imbalance.

Table S15: Top 10 HP for **HCC Adaptive NTCP** with **GLoGD-GNN**

HP #	*Opt lr mu*	*Opt lr T*	*Node*	*Epoch*	*Training AUC*		*Avg Validation AUC*	
					Mean	*Stdev*	*Mean*	*Stdev*
**1**	**0.0001**	**0.0005**	**256**	**100**	**0.98**	**0.02**	**0.68**	**0.21**
2	0.0001	0.0005	128	200	0.94	0.06	0.66	0.14
3	0.0001	0.001	256	200	0.99	0.00	0.65	0.23
4	0.0005	0.0001	64	100	0.98	0.02	0.64	0.23
5	0.0001	0.001	64	400	0.93	0.07	0.63	0.19
6	0.0001	0.001	128	200	0.95	0.05	0.62	0.18
7	0.0001	0.0005	256	200	0.86	0.22	0.61	0.20
8	0.0001	0.001	128	400	0.88	0.21	0.61	0.20
9	0.0005	0.0001	128	100	0.78	0.23	0.61	0.21
10	0.0005	0.0001	64	400	0.91	0.17	0.61	0.26

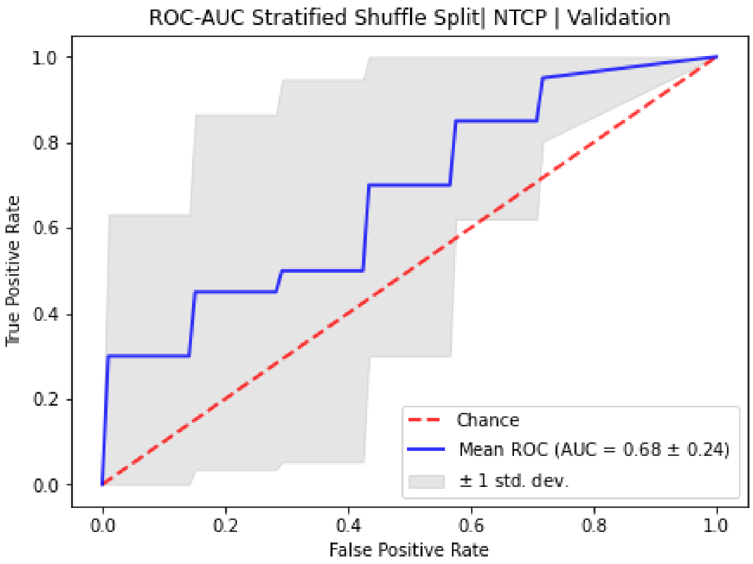

Figure S25: 10-fold stratified shuffle 80-20 split ROC for HCC Non-Adaptive RTOE of NTCP modeled with GLoGD-GNN architecture.

S8.4 Synthetic Patient Generation via WGAN-GP

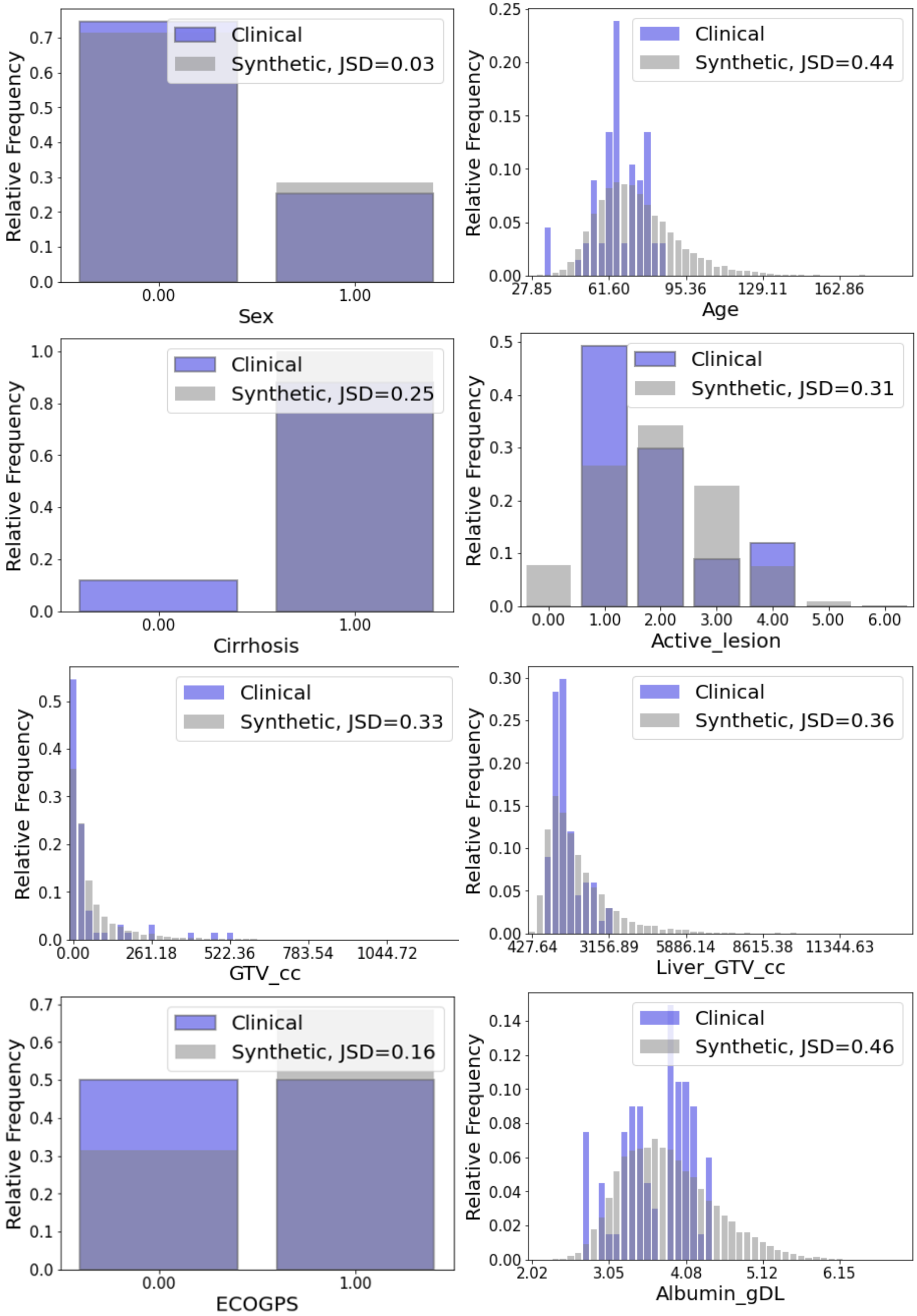

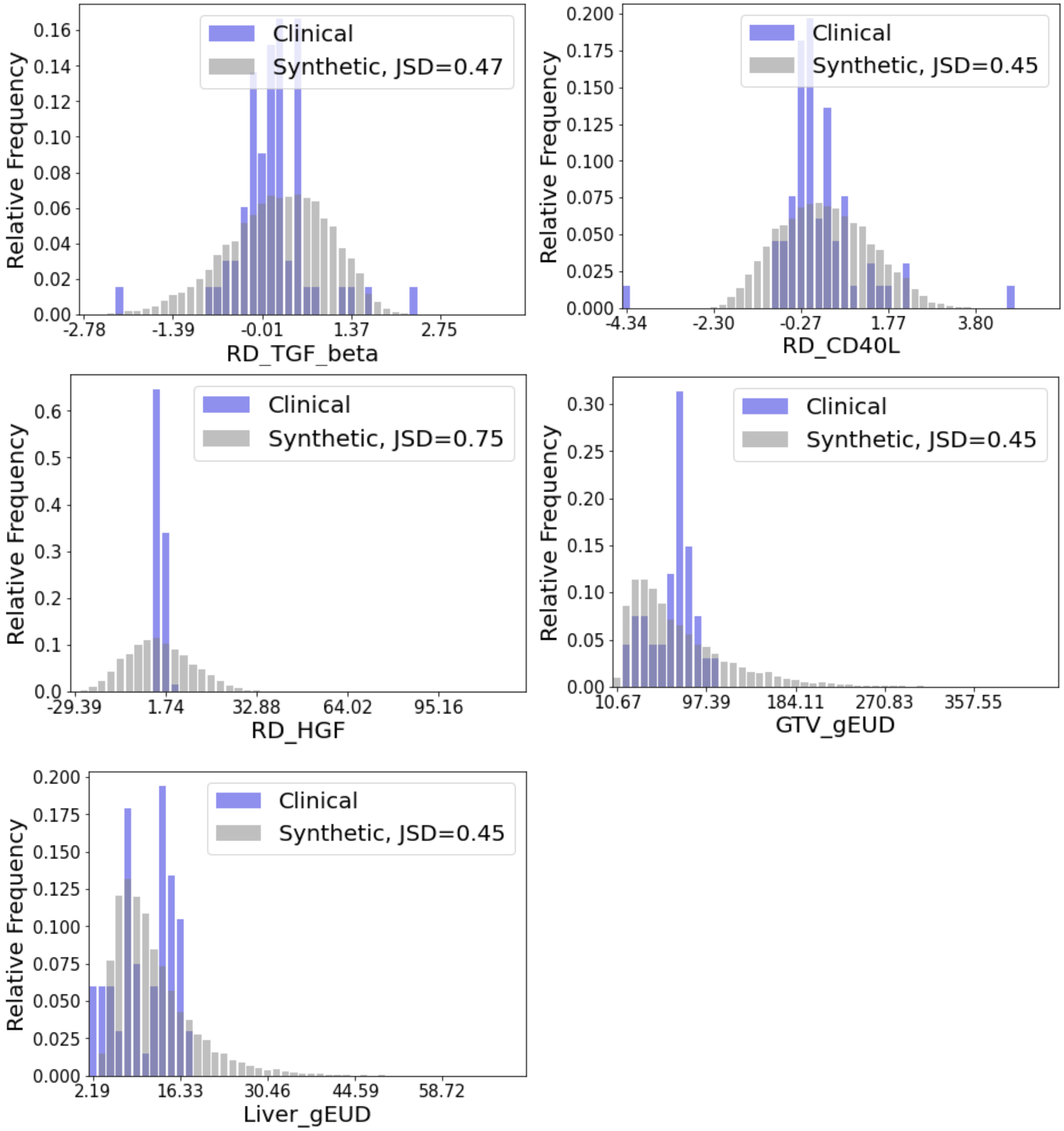

Figure S26. Distribution comparison of generated and original HCC dataset. Jensen Shannon Divergence metric between the distributions is provided for further insight on the differences.

S8.5 ODM Decision Analysis

Table S16: ODM Decision Analysis Results for HCC

Error [Gy/frac]	**Overall (n=64)**		**Positive Clinical Outcome (n=54 (83%))**		**Negative Clinical Outcome (n=10 (15%))**	
	RMSD	MAD	RMSD	MAD	RMSD	MAD
**Single GNN RTOE + DDQN ODM**	4.75 ± 0.16	3.69 ± 0.16	4.25 ± 0.26	3.34 ± 0.22	6.78 ± 0.35	5.60 ± 0.38
**GloGD GNN ROTE + DDQN ODM**	2.96 ± 0.42	2.31 ± 0.47	2.79 ± 0.50	2.10 ± 0.54	4.02 ± 0.23	3.46 ± 0.17

Self-Evaluation [Count (%)]	**Overall (n=64)**			**Positive Clinical Outcome (n=54 (83%))**		**Negative Clinical Outcome (n=10 (15%))**	
	Good	Bad	Not Sure	Good	Not Sure	Good	Bad
**Single GNN RTOE + DDQN ODM**	15 (23%)	9 (14%)	40 (62%)	14 (26%)	40 (74%)	1 (10%)	9 (90%)
**GloGD GNN ROTE + DDQN ODM**	30 (46%)	7 (11%)	27 (42%)	27 (50%)	27 (50%)	3 (30%)	7 (70%)

Error ± SEM | Error between average recommendation and clinical decisions| ensemble of 5 models

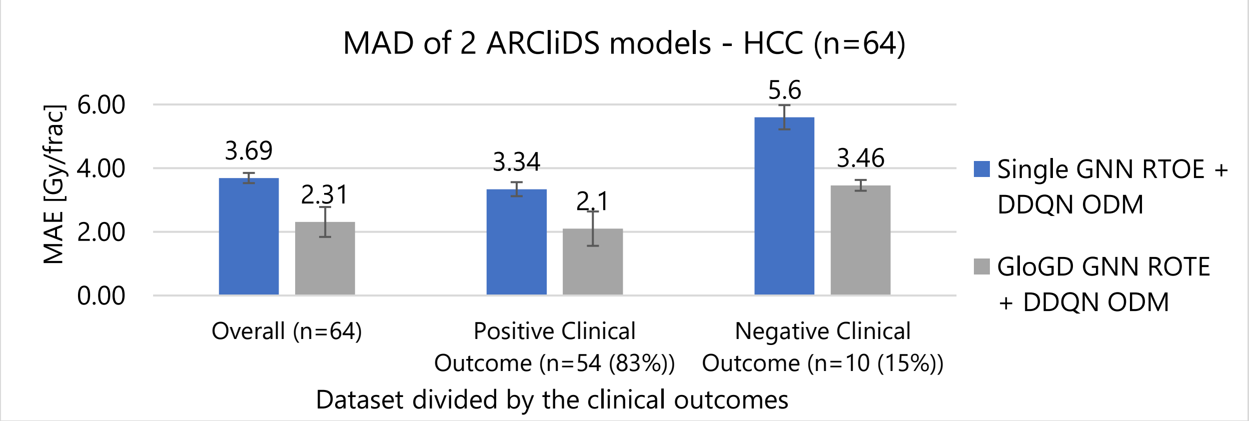

Figure S27: Mean Absolute Difference (MAD) of ARCliDS's two model architecture for HCC patients grouped together according to the outcomes.

S8.5.1 DDQN trained on Single GNN RTOE – HCC

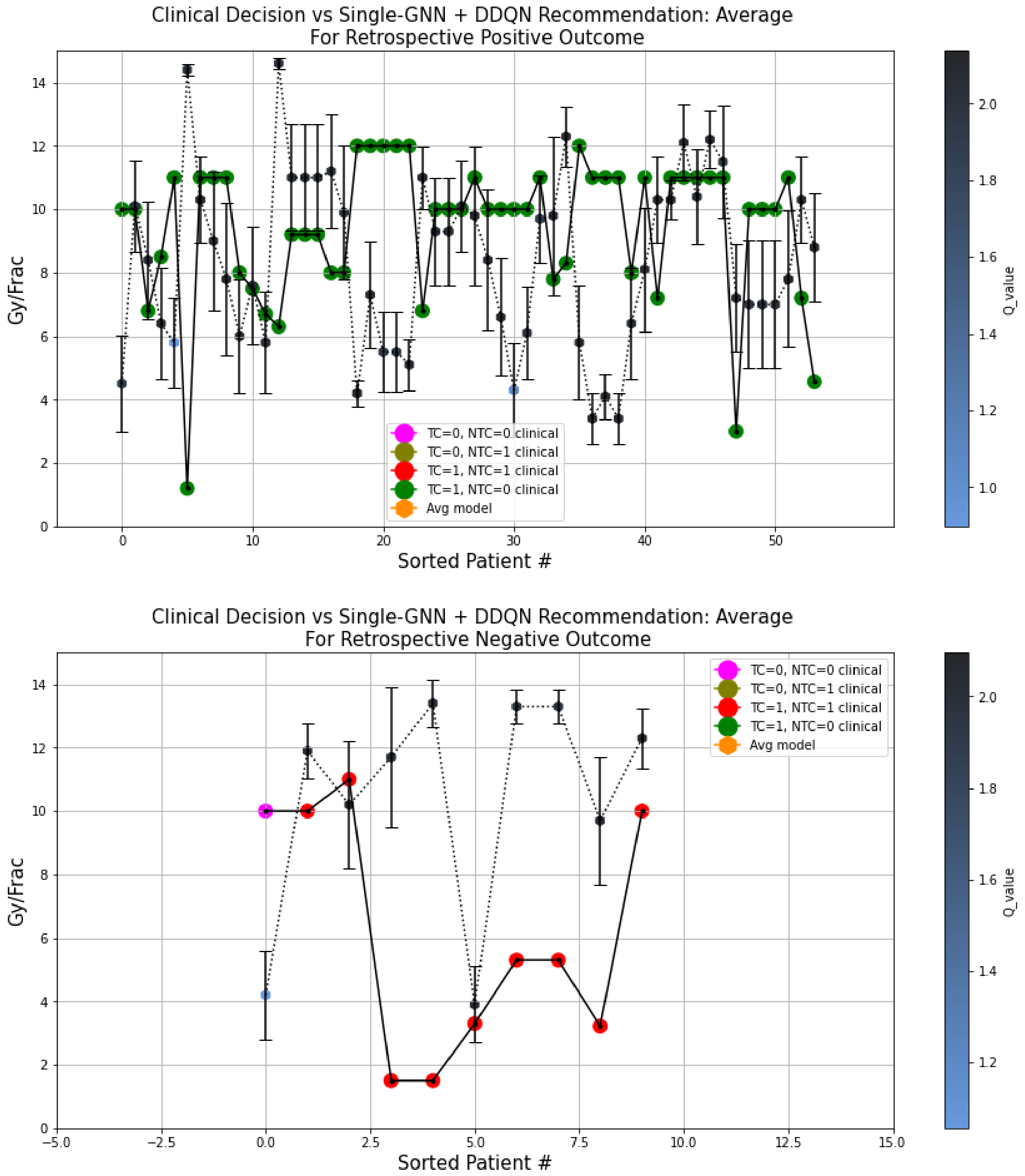

Figure S28: A visual comparison between the AI recommendation generated by the Single-GNN RTOE + DDQN ODM architecture and clinical decision for 2 groups of HCC patients divided according to the clinical outcomes. The clinical decisions are color coded with the outcomes and the ARCliDS recommendations are color coded with the respective q-values. Qualitatively, the q-value can be considered as the AI confidence in its recommendations.

S8.5.2 DDQN trained on GLoGD-GNN RTOE - HCC

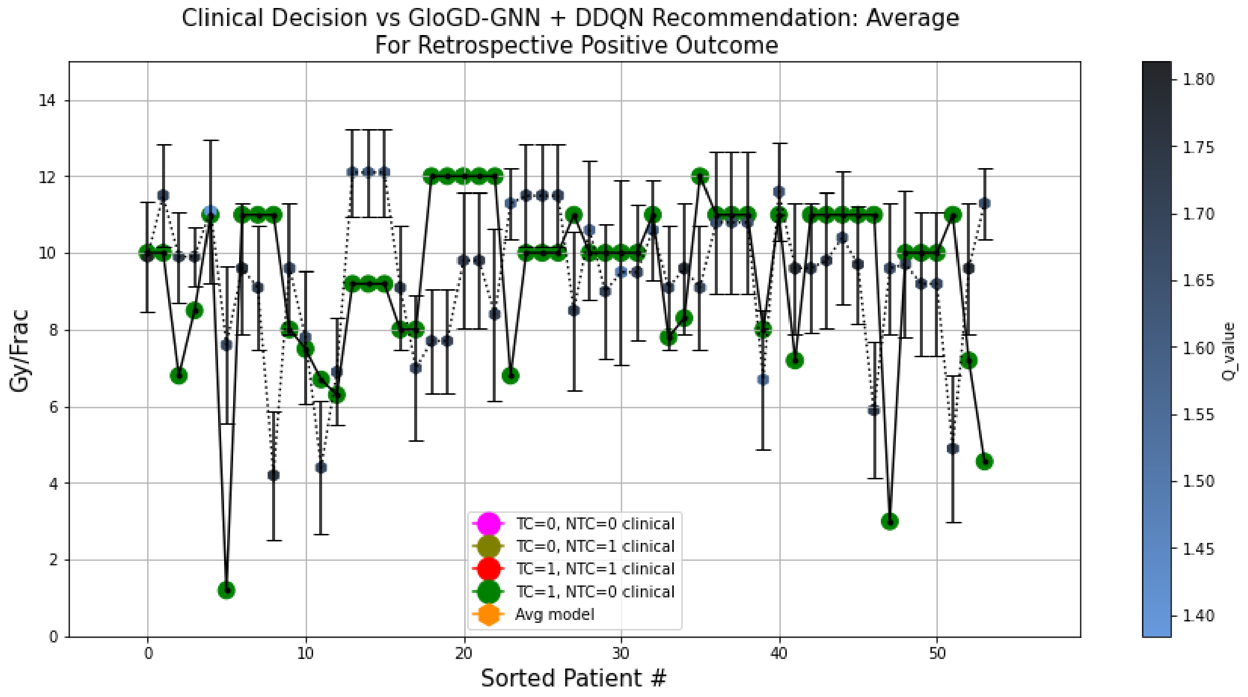

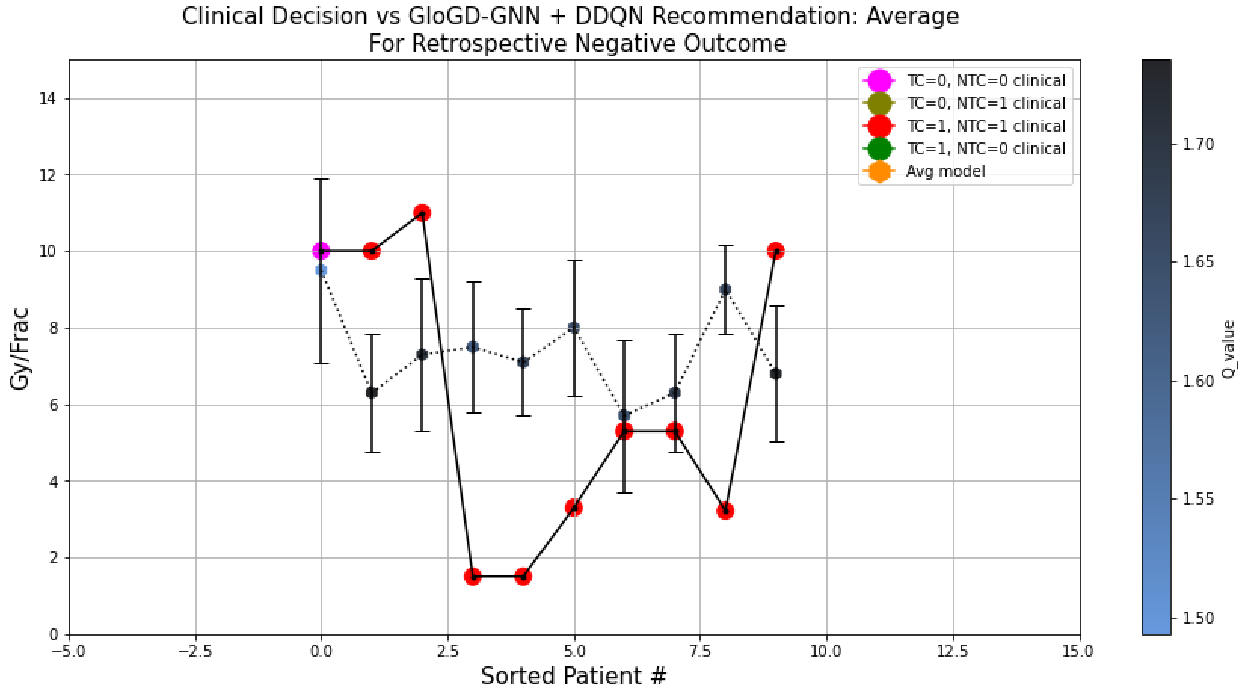

Figure S29: A visual comparison between the AI recommendation generated by the GloGD-GNN RTOE + DDQN ODM architecture and clinical decision for 2 groups of HCC patients divided according to the clinical outcomes. The clinical decisions are color coded with the outcomes and the ARCliDS recommendations are color coded with the respective q-values. Qualitatively, the q-value can be considered as the AI confidence in its recommendations.

S8.5.3 Experimentation with population-based reward goal for HCC

Due to data-related issues, as described in the Discussion Section S10, both TCP and NTCP response estimated by the GloGD-GNN ROTE were flatter than expected. Due to a flatter NTCP response, which did not span the whole probability space, the population-based reward goal severely limited the AI dose recommendation. In this section, we present plots for three different reward goals. As seen from the plots, for ntcp < 25%, the AI recommended lower dose values. The best recommendation corresponded to tcp > 90% and ntcp < 40%, however, the overall RMSD was lower than tcp > 50% and ntcp <50%.

1.

$$r_{HCC} = \begin{cases} R+2, & if\ tcp > 0.90\ \ and\ ntcp < 0.25 \\ R+1, & if\ tcp > 0.50\ \ and\ ntcp < 0.50 \\ R, & otherwise \end{cases}$$

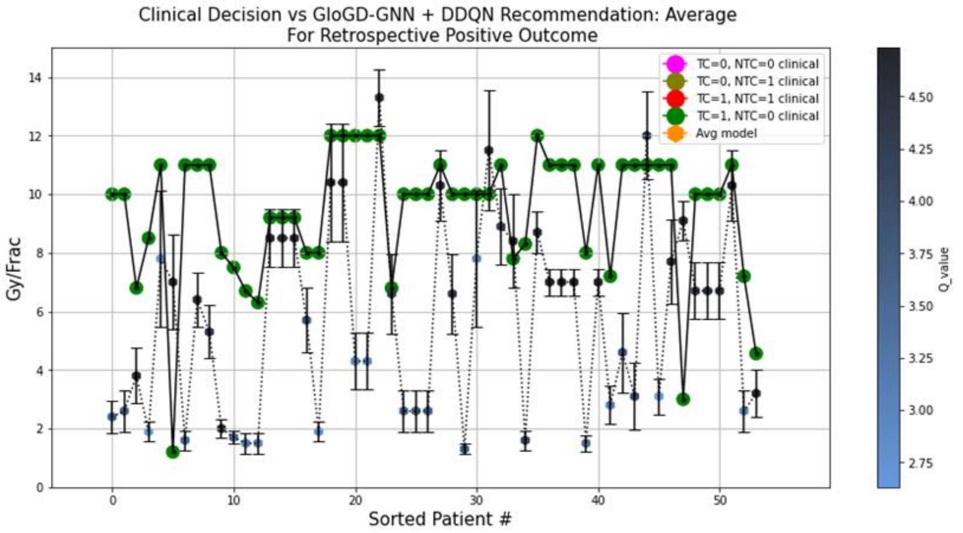

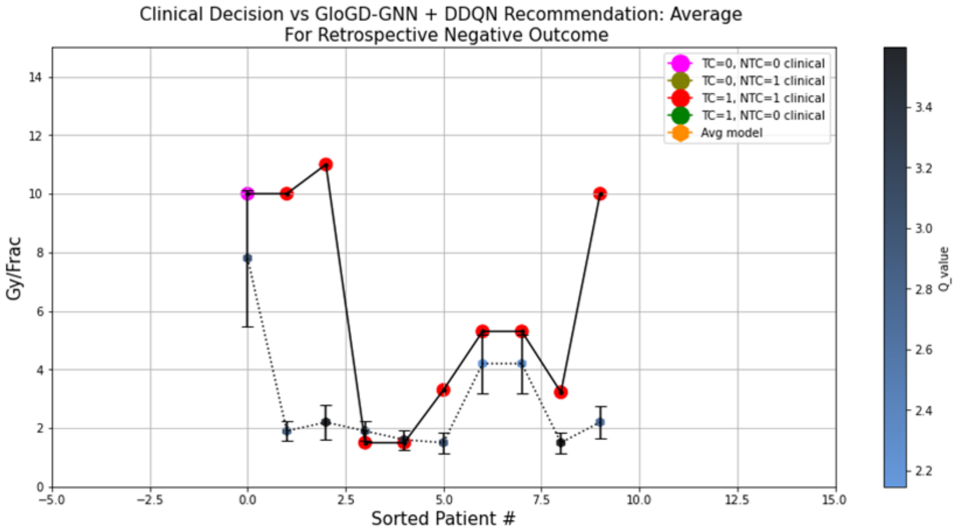

Figure S30: A visual comparison between the AI recommendation generated by the GloGD-GNN RTOE + DDQN ODM architecture and clinical decision for 2 groups of HCC patients divided according to the clinical outcomes. The ODM was trained with population-based reward goal of tcp > 90% and ntcp < 25%.

2.

$$r_{HCC} = \begin{cases} R+2, & if\ tcp > 0.50\ \ and\ ntcp < 0.25 \\ R+1, & if\ tcp > 0.50\ \ and\ ntcp < 0.50 \\ R, & otherwise \end{cases}$$

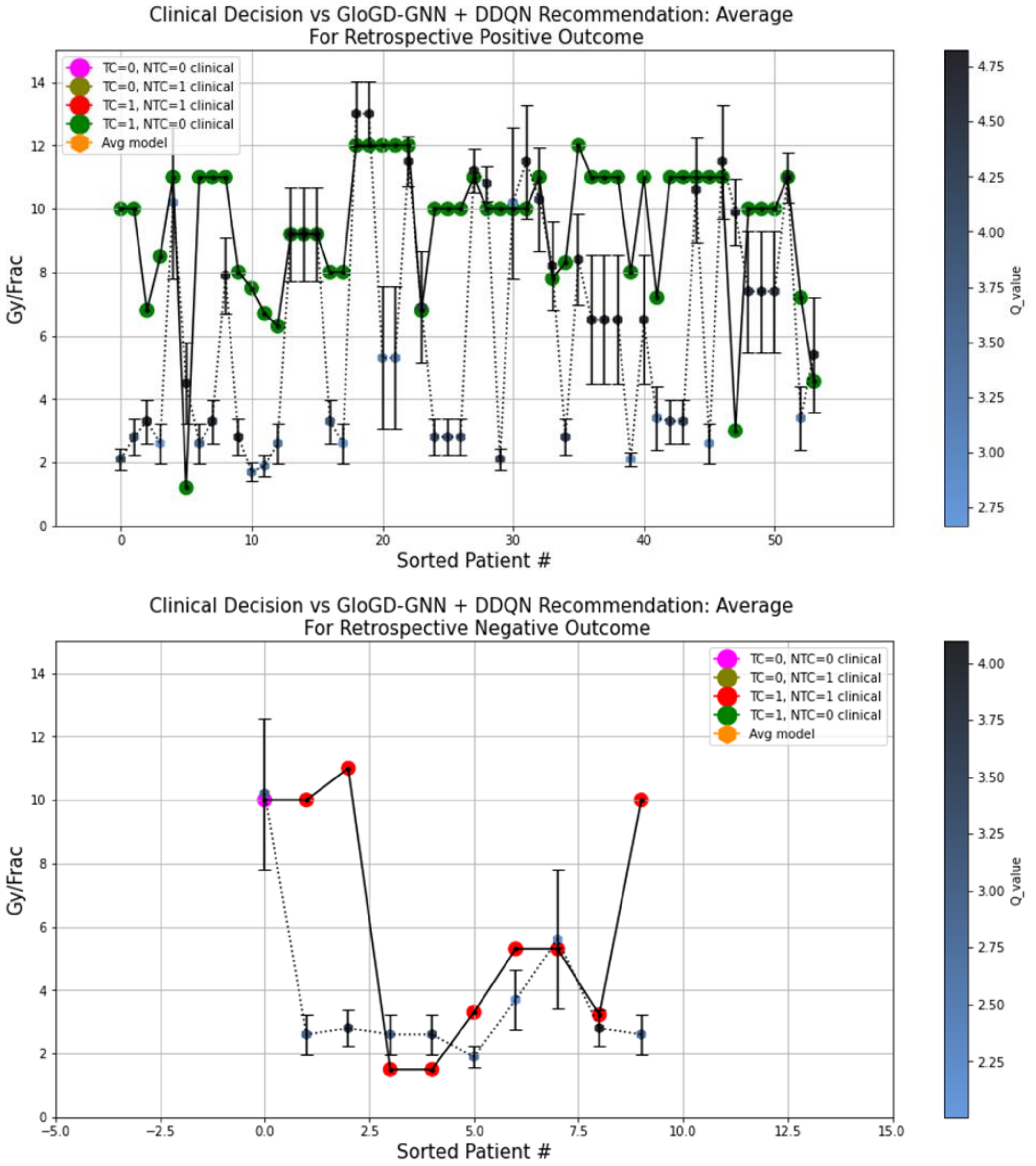

Figure S31: A visual comparison between the AI recommendation generated by the GloGD-GNN RTOE + DDQN ODM architecture and clinical decision for 2 groups of HCC patients divided according to the clinical outcomes. The ODM was trained with population-based reward goal of tcp > 50% and ntcp < 25%.

3.

$$r_{HCC} = \begin{cases} R+2, & if\ tcp > 0.90\ \ and\ ntcp < 0.40 \\ R+1, & if\ tcp > 0.50\ \ and\ ntcp < 0.50 \\ R, & otherwise \end{cases}$$

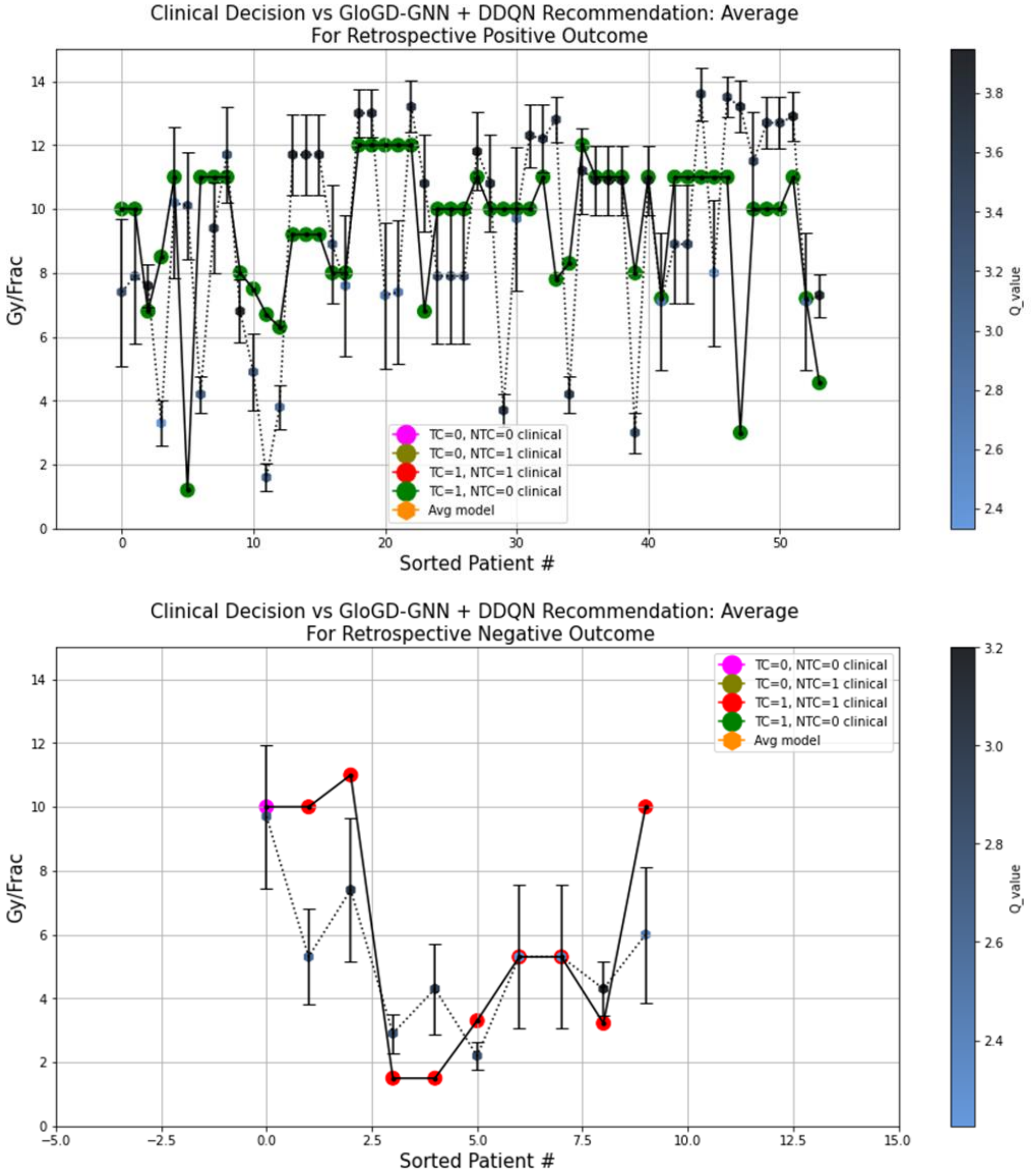

Figure S32: A visual comparison between the AI recommendation generated by the GloGD-GNN RTOE + DDQN ODM architecture and clinical decision for 2 groups of HCC patients divided according to the clinical outcomes. The ODM was trained with population-based reward goal of tcp > 90% and ntcp < 40%.

References

1. Luo, Y. *et al.* A multiobjective Bayesian networks approach for joint prediction of tumor local control and radiation pneumonitis in nonsmall-cell lung cancer (NSCLC) for response-adapted radiotherapy. *Med Phys* **45**, 3980–3995 (2018).

2. Hamilton, W. L. *Graph Representation Learning*. vol. 14 (Morgan and Claypool, 2020).

3. *A Guide to Outcome Modeling In Radiotherapy and Oncology : Listening to the Data*. (CRC Press, 2018).

4. Ramírez, M. F., Huitink, J. M. & Cata, J. P. Perioperative Clinical Interventions That Modify the Immune Response in Cancer Patients. *Open J Anesthesiol* **03**, 133–139 (2013).

5. Schaue, D., Kachikwu, E. L. & McBride, W. H. Cytokines in Radiobiological Responses: A Review. *Radiat Res* **178**, 505–523 (2012).

6. Warltier, D. C., Laffey, J. G., Boylan, J. F. & Cheng, D. C. H. The Systemic Inflammatory Response to Cardiac Surgery. *Anesthesiology* **97**, 215–252 (2002).

7. Mahasittiwat, P. *et al.* Metabolic tumor volume on PET reduced more than gross tumor volume on CT during radiotherapy in patients with non-small cell lung cancer treated with 3DCRT or SBRT. *J Radiat Oncol* **2**, 191–202 (2013).

8. Carrier-Vallieres, M. Radiomics: enabling factors towards precision medicine. (McGill University , 2018).

9. *A Guide to Outcome Modeling In Radiotherapy and Oncology : Listening to the Data*. (CRC Press, 2018).

10. Hildebrandt, M. A. T. *et al.* Genetic Variants in Inflammation-Related Genes Are Associated with Radiation-Induced Toxicity Following Treatment for Non-Small Cell Lung Cancer. *PLoS One* **5**, e12402 (2010).

11. Chang, J. S. *et al.* Nucleotide excision repair genes and risk of lung cancer among San Francisco Bay Area Latinos and African Americans. *Int J Cancer* **123**, 2095–2104 (2008).

12. Kiyohara, C. & Yoshimasu, K. Genetic polymorphisms in the nucleotide excision repair pathway and lung cancer risk: A meta-analysis. *Int J Med Sci* 59–71 (2007) doi:10.7150/ijms.4.59.

13. Nagpal, N. & Kulshreshtha, R. miR-191: an emerging player in disease biology. *Front Genet* **5**, (2014).

14. Ricciuti, B. *et al.* Non-coding RNAs in lung cancer. *Oncoscience* **1**, 674–705 (2014).

15. Lin, S. & Gregory, R. I. MicroRNA biogenesis pathways in cancer. *Nat Rev Cancer* **15**, 321–333 (2015).

16. Caraceni, P., Tufoni, M. & Bonavita, M. E. Clinical use of albumin. *Blood Transfus* **11 Suppl 4**, s18-25 (2013).

17. Morikawa, M., Derynck, R. & Miyazono, K. TGF-β and the TGF-β Family: Context-Dependent Roles in Cell and Tissue Physiology. *Cold Spring Harb Perspect Biol* **8**, (2016).

18. Elgueta, R. *et al.* Molecular mechanism and function of CD40/CD40L engagement in the immune system. *Immunol Rev* **229**, 152–172 (2009).

19. Perreau, M. *et al.* The cytokines HGF and CXCL13 predict the severity and the mortality in COVID-19 patients. *Nat Commun* **12**, 4888 (2021).

20. Matsumoto, K. & Nakamura, T. Roles of HGF as a pleiotropic factor in organ regeneration. *EXS* **65**, 225–49 (1993).
